# A Design Framework for Microintervention Software Technology in Digital Health: Critical Interpretive Synthesis

**DOI:** 10.2196/72658

**Published:** 2025-09-12

**Authors:** Dan Roland Persson, Mel Ramasawmy, Nushrat Khan, Amitava Banerjee, Ann Blandford, Jakob Eyvind Bardram, Per Bækgaard

**Affiliations:** 1 Department of Computer Science University of Copenhagen Copenhagen N Denmark; 2 Department of Applied Mathematics and Computer Science Technical University of Denmark Kongens Lyngby Denmark; 3 Institute of Health Informatics University College London London United Kingdom; 4 Wolfson Institute of Population Health Queen Mary University of London London United Kingdom; 5 Department of Primary Care and Public Health Faculty of Medicine Imperial College London London United Kingdom; 6 University College London Interaction Centre University College London London United Kingdom; 7 Department of Health Technology Technical University of Denmark Kongens Lyngby Denmark

**Keywords:** microinterventions, narrative, framework, personalization, digital health, microintervention, micro-interventions, micro interventions

## Abstract

**Background:**

Although many digital health interventions have been studied in human-computer interaction, long-term engagement remains a challenge. Microinterventions that leverage small but meaningful steps toward health outcomes have been proposed as a method for providing flexible and engaging, yet short and focused, interventions. One way to mitigate the potentially limiting scope of each microintervention is through narratives combining multiple different microinterventions over time to facilitate greater effects and engagement. As an emerging field of research, significant focus has thus far been placed on the creation and testing of individual microinterventions with little consideration for the consolidated experience, that is, the narrative over time. A more concise terminology and framework could make it easier to design, evaluate, and compare systems for microinterventions, facilitate collaboration in the field, and foster communication across required disciplines.

**Objective:**

This paper aims to provide a framework for the creation of microintervention systems and the narratives served by these through their microinterventions.

**Methods:**

To synthesize a framework, we carried out a critical interpretive synthesis involving reciprocal translational analysis, refutational synthesis, and lines-of-argument synthesis, based on a systematic review of microintervention literature following the PRISMA (Preferred Reporting Items for Systematic Reviews and Meta-Analyses) guidelines across the Association for Computing Machinery Digital Library, PubMed, Scopus, and Web of Science. To assess the resulting framework, we applied it retroactively back onto the literature.

**Results:**

Based on a review including 35 studies, of which 80% (n=28) were published after 2020, we present and rationalize the Design for Microintervention Software Technology (D-MIST) framework, which can be used to better classify, design, and evaluate microinterventions. D-MIST defines a microintervention system on three levels: (1) the narrative, (2) the microintervention, and (3) the event. The narrative is the consolidated experience of engaging with multiple microinterventions over time, supporting a narrative goal, for example, a clinical goal. A microintervention is a highly focused intervention consisting of self-contained events aiming to facilitate small but meaningful steps toward the narrative goal. The event attempts a positive momentary change through a resource, for example, an exercise. Events may, for example, be designed for single use, present variations to facilitate engagement, or present order-dependent sequential resources. We additionally discuss supporting constructs, such as decision rules, conceptual models, and the system’s interaction model, that is, how the system interacts with the user through its design and other actors to create a consolidated narrative.

**Conclusions:**

The resulting D-MIST framework, based on current state-of-the-art literature, describes the components of microintervention systems and how these contribute to a consolidated experience. Based hereon, we suggest opportunities for personalization of microintervention systems, discuss design guidelines, and the roles of actors in creating health narratives aiming to obtain better distal health outcomes.

## Introduction

### Background

Digitization has enabled many new approaches in digital health technology with a range of apps supporting mental and physical conditions [1-5]. Although many digital health interventions for managing health conditions have been designed and evaluated, there is growing evidence that long-term engagement is typically challenged, as also reflected by high attrition [6-8]. Reasons for this include gaps in (1) understanding how people fit digital interventions into their lives [6,9] and (2) making interventions personally and contextually relevant [10,11]. Both issues address the core challenge of how digital interventions can remain engaging over time [9,10] and hence are likely to have a meaningful long-term health outcome. Further, users may expect interventions to be quick and convenient [12], suggesting a need for flexibility, contextual adaptation, and personalization [13].

One approach to better fit digital interventions into everyday life is through the use of microinterventions, which are brief interventions aiming to have a positive effect on targeted symptoms [14]. Microinterventions differ from traditional interventions by being short and highly focused [14], delivered to help users achieve their goals [12]. Paredes et al [15], for example, used exercises, games, and videos to elicit momentary stress reductions upon user request. A shorter length aims to reduce barriers to initial and sustained engagement, meeting users’ often limited capacity or willingness to invest efforts into interventions [12].

As an emerging field of research, significant emphasis has been placed on the investigation of selected microinterventions and their effects on targeted symptoms and measures. Studies in this domain have shown microinterventions to improve mood [16,17], stress [15], promote physical activity [18], and support chronic illness [19,20]. If digital interventions are to be both usable and (clinically) effective, they need to be evaluated over different timescales—both proximal and distal. Proximal outcomes are those that can be evaluated within a short timeframe (immediately or within hours), such as event effects [12] and early engagement. Distal outcomes are those that can only be evaluated after an extended timeframe (weeks or months), including long-term engagement and changes in health outcomes.

Given each microintervention’s relatively short and focused nature, the term “microintervention care” has been suggested as a way of describing the larger therapeutic process where several interventions are used in combination for a greater effect [12]. To make microinterventions personally and contextually relevant, a key approach is to construct a narrative, which is an “account of connected events” [21], combining several microinterventions. The true potential of microinterventions may be realized using these as building blocks for larger narratives, with individual microinterventions’ effects contributing to the narrative’s therapeutic goals rather than being purely isolated interventions. Schroeder et al [22], for example, create a narrative of 5 microinterventions, each comprising several events, with a narrative goal to develop skills that help users maintain positive relationships. One included microintervention is “Emotional Regulation,” intended to help control emotions, in turn containing events like “Understand Emotions” and “Change Skills” [22].

However, when investigating the reported studies on microinterventions, it appears that the terminology involved in describing what constitutes microinterventions varies subtly or even significantly, with no unified definition of the terms, what microinterventions consist of, and how they are delivered. This hampers the design and optimization of microinterventions as part of technologies, while also making it more difficult to assess, evaluate, and compare which microintervention components are most effective and under what circumstances. Thus, while it is possible to assess and compare the outcome of a microintervention on an overall level, it is difficult to understand what components of the microintervention made the difference. Often, such digital health intervention studies are treated as a “black box,” where it is difficult to precisely see what constitutes the intervention. For example, if comparing two different smoking cessation studies, one showing better results than the other, it would be relevant to investigate and define exactly which components of the intervention actually were behind the two approaches, including what health narrative was used, which specific microintervention steps were suggested, and how the effect of such intervention steps was assessed and used.

A common terminology is essential in order to understand, discuss, and compare microintervention studies, thereby promoting interdisciplinary collaboration and communication [23]. Researchers could also benefit from a better and more precise understanding of the complex literature [24], including available building blocks, collections, or “catalogs” of interventions, their events, and when to best make use of these. Moreover, an improved understanding of the user experience may positively impact the uptake and efficacy of such microinterventions.

### Related Work

#### Microinterventions

Research on microinterventions as a specific field of study has been steadily increasing in recent years. Modern interest in microinterventions within computer science seems to have picked up from 2014, with early works [15,19] such as that by Paredes et al [15], describing a system recommending contextually relevant microinterventions from a library of 16 possible microinterventions. Outside the field of computer science, the term microintervention has seen use by, for example, Wetzel [25] and Rinne et al [26]. Rinne et al [26] used the term to denote “brief activities conducted by teachers in which the presentation of numerical information is preceded and/or followed by particular exercises that scaffold students’ decision-making procedures,” although variations of this definition can also be found elsewhere [27,28].

In 2019, Fuller-Tyszkiewicz et al [14] provided a broad definition of what microinterventions are, and in 2020, Baumel et al [12] further conceptualized the idea of “microintervention care” as consisting of narratives of microinterventions facilitated by a “hub,” for example, a digital app or coach [12]. Arguably, works both prior to and after Fuller-Tyszkiewicz et al’s [14] definition can be classified as microintervention systems. For example, Quinn et al [29] describe a system providing feedback messages from a library of 1000, similar to the approaches used in recent microintervention systems [18,30]. Other systems have used microinterventions to reduce sedentary time [31], motivate short exercise sessions [32], provide personalized feedback [33], or reduce stress through breathing exercises [34]. Outside of health and well-being, the term microintervention has also seen some use in game research [35,36].

#### Intervention Patterns and Microinterventions

Generally, microinterventions use previously established and grouped behavior change techniques (BCTs) [37] such as shaping knowledge [38], comparisons of outcomes [39], and feedback and monitoring [20]. Many proposed microinterventions use multiple different BCTs [14,20,40] in line with commercially deployed app-interventions [41]. More broadly, digital technologies aiming to support behavior change are referred to as digital behavior change interventions (DBCIs) [8]. Various frameworks and models supporting DBCIs development have been suggested, such as the Just-In-Time Adaptive Interventions (JITAI) framework [23]. Mohr et al’s [42] BIT model provides a framework for articulating the relationship between intervention aims, characteristics, workflow, and implementation. However, prior works have also noted some difficulty in translating BCTs to a digital context, particularly regarding engagement [8,43]. Fulton et al [43] suggest co-design or cocreation [44] as a means of improving engagement. Mummah et al’s [45] “IDEAS” DBCI design framework highlights empathizing with users before attempting to ground desired target behaviors in behavioral theory.

In the past decade, many intervention designs have been conceptualized, proposed, and explored. According to Fuller-Tyszkiewicz et al [14], microinterventions, just-in-time (JIT), JITAI, and ecological momentary interventions (EMIs) may be differentiated from standard intervention packages in terms of the depth of content and timing of content delivery. Heron and Smyth [46] describe EMI as: “...provid[ing] a framework for treatments characterized by the delivery of interventions to people as they go about their daily lives.” JITAI has previously been described as consisting of two concepts: JIT and adaptive [23]. JIT “is used to describe an attempt to provide the right type (or amount) of support, at the right time, namely neither too early nor too late” and “adaptation is defined as the use of ongoing (dynamic) information about the individual to modify the type, amount, and timing of support” (Nahum-Shani et al [23]).

Microinterventions can be differentiated from other designs by their exclusive focus on shorter and more focused interventions that can be “quickly” consumed and have an immediate impact on targeted symptoms [12,14]. Broadly, microinterventions aim to meet users’ often limited capacity or willingness to invest effort in beneficial therapeutics [12]. Microinterventions, in particular, can be differentiated from the JITAIs described by Nahum-Shani et al [23] in that these often rely on users’ subjective judgment rather than more well-defined protocols handled by systems. As such, many key constructs described by Nahum-Shani et al [23], like “Just-in-time support,” “Adaptation,” “Intervention options,” and “Decision rules,” are neither used broadly nor universally present.

While digital microinterventions are generally EMIs [47] and overlap with JIT and JITAI in the sense that some systems use these patterns [11,23], this is not universally the case. Some microintervention systems allow users to choose when and which microintervention to engage with [14,48], counter to JITAIs, where decisions are based on the intervention protocol, and to JIT, as interventions are not necessarily deployed at the right time [23]. Further, while JITAIs may include increasing or decreasing intensity or transitioning to other therapy types [11], microinterventions, given their short, highly focused nature, may result in users not receiving interventions for periods of time [12].

### Objective

This paper aims to provide a framework for the creation of microintervention systems and the narratives served by these through their microinterventions. This framework is intended to be an evidence-based conceptual framework for classifying, designing, and studying microinterventions in digital health technologies that address different health conditions. The framework is useful for designers and researchers wishing to build or study microintervention systems. The framework is based on a review of the literature and the critical interpretive synthesis (CIS) method [24]. The main contribution is the Design for Microintervention Software Technology (D-MIST) conceptual framework for the design and assessment of microintervention technology. This framework defines the core components of microintervention systems and provides a classification using concepts, evidence, and examples from the reviewed literature. The framework defines core concepts like the “microintervention system,” “narrative,” “microintervention,” and “event.” The framework can help design and evaluate microintervention technology by providing more precise and evidence-based definitions. Based on the D-MIST framework, we discuss the role of actors in the decision-making process, personalization, and present a catalog of microinterventions.

## Methods

### Study Design

Development of the D-MIST framework is based on the CIS method [24]. This approach was motivated by several factors: CIS offers an alternative to a standard systematic review, which usually focuses on producing an aggregative overview of literature by instead creating an interpretive synthesis [24]. CIS is particularly useful in this case as key characteristics (concepts or components) are not well specified nor used consistently in the literature. Prior to this work, for example, no definition existed for “microintervention systems,” including the system’s role, with the term “microintervention” seeing varied uses in the literature [12,14,15] with some works suggesting these are each one piece of content attempting change [15,20,49] and others seeing each microintervention as consisting of one or multiple events [12,14]. Through its exploratory and interpretive nature, CIS supports the development of concepts and theories: in this case, framework development using induction and interpretation [24]. This flexibility also accommodates and indeed encourages including diverse study types [24,50,51]; this is also useful given the early-stage nature of microintervention research that includes many design, feasibility, and pilot studies with relatively short study durations and limited participants.

One limitation of the CIS methodology is that it does not include a formal search protocol; accordingly, we have followed PRISMA (Preferred Reporting Items for Systematic Reviews and Meta-Analyses) guidelines [52]. However, the focus of this paper is not on providing a systematic aggregate overview of the literature, but rather on presenting the synthesized framework, including a narrative overview of the literature, which leads to the framework rationale.

### Review

#### Search Strategy

This work is scoped on the literature, which uses the term “microintervention.” This avoided us imposing a definition of “microintervention” and “microintervention system.” Moreover, we wanted to avoid limiting the initial search results to a certain context, that is, “mHealth” or “digital health,” to capture the broadest possible view on the subject area. The search terms used were: “micro intervention” OR “micro-intervention” OR “micro interventions” OR “micro-interventions.” The full search strategy can be found in Multimedia Appendix 1.

Four databases were searched: the Association for Computing Machinery (ACM) digital library, Scopus, PubMed, and Web of Science—all databases well suited for this type of conceptual synthesis [53]. The search fields for ACM, PubMed, and Web of Science were “all fields” (“anywhere” on ACM); for SCOPUS, it was limited to “Article Title, Abstract, and Keywords.” No filters were applied to any database.

#### Eligibility Criteria

Inclusion criteria were: (1) a peer-reviewed journal study or conference paper, (2) written in English, (3) describing the implementation of at least one microintervention or key components of such, (4) targeting adults, and (5) relating to mental or physical health or well-being. Studies were excluded if (1) they did not focus on health or well-being, (2) participants were nonadults, or (3) the paper did not describe the implementation of at least one microintervention or key components of these. Moreover, (4) non-English texts and (5) papers such as reviews, protocols, opinion pieces, commentaries, perspectives, and books were removed.

#### Study Selection

The documented review and analysis were conducted on June 10, 2024, and were subsequently validated on November 11, 2024, by two authors. Duplicates were removed by the first author. Screening of records by title and abstract was conducted independently by two authors, with an adjudication process used for disagreements, which included discussion and rereviewing. Full-text screening against inclusion or exclusion criteria was conducted by one author, with an additional author cross-checking these.

#### Data Extraction

Data extraction initially consisted of overall study characteristics extracted into an Excel (Microsoft Corp) template, for example, paper type, targets, users, and duration. Additional data extraction consisted of document highlighting, paragraph extraction, and thematic organization in line with the CIS approach [24], eventually resulting in the framework itself and additional extraction of microinterventions, event types, number of events, and used resources (eg, text or video) based on the framework.

#### Quality Appraisal

As noted in the motivation for our methodological approach, we did not omit any studies based on quality issues and accordingly did not use any structured tool to appraise studies.

### CIS

Following the approach presented by Dixon-Woods et al [24], three of the authors engaged in the CIS approach involving three strategies: (1) reciprocal translational analysis, (2) refutational synthesis, and (3) lines-of-argument synthesis.

(1) In our reciprocal translational analysis, key definitions, descriptions, and uses were initially identified, compared, and translated into one another. Maps and initial models were created based on the presented descriptions and uses. (2) In refutational synthesis, contradictions between descriptions were identified and attempts were made to explain these through the available literature, including a critical and reflexive approach to the literature insights and scrutinizing arguments, evidence, and theory. (3) In lines-of-argument, we built our initial framework interpretation based on findings from the literature and prior phases. This included integrating evidence from across studies into a coherent framework and generating new “synthetic constructs” (components). These components are grounded in evidence and result from an overall interpretation of available evidence, allowing different definitions and uses to be unified. Our framework section on microinterventions, for example, presents an argument synthesis for a unified definition of microinterventions based on previous definitions, uses, and other components.

The validity of the synthesis is based on the CIS approach, by retroactively applying the framework back onto the literature and through the experience of the research team, which covers multiple disciplines, including human-computer interaction, user experience, computer science, and (digital) health. The process spanned two countries and included multiple levels of seniority and experience in both academia and industry.

## Results

### Study Selection and Characteristics

**Figure 1:**
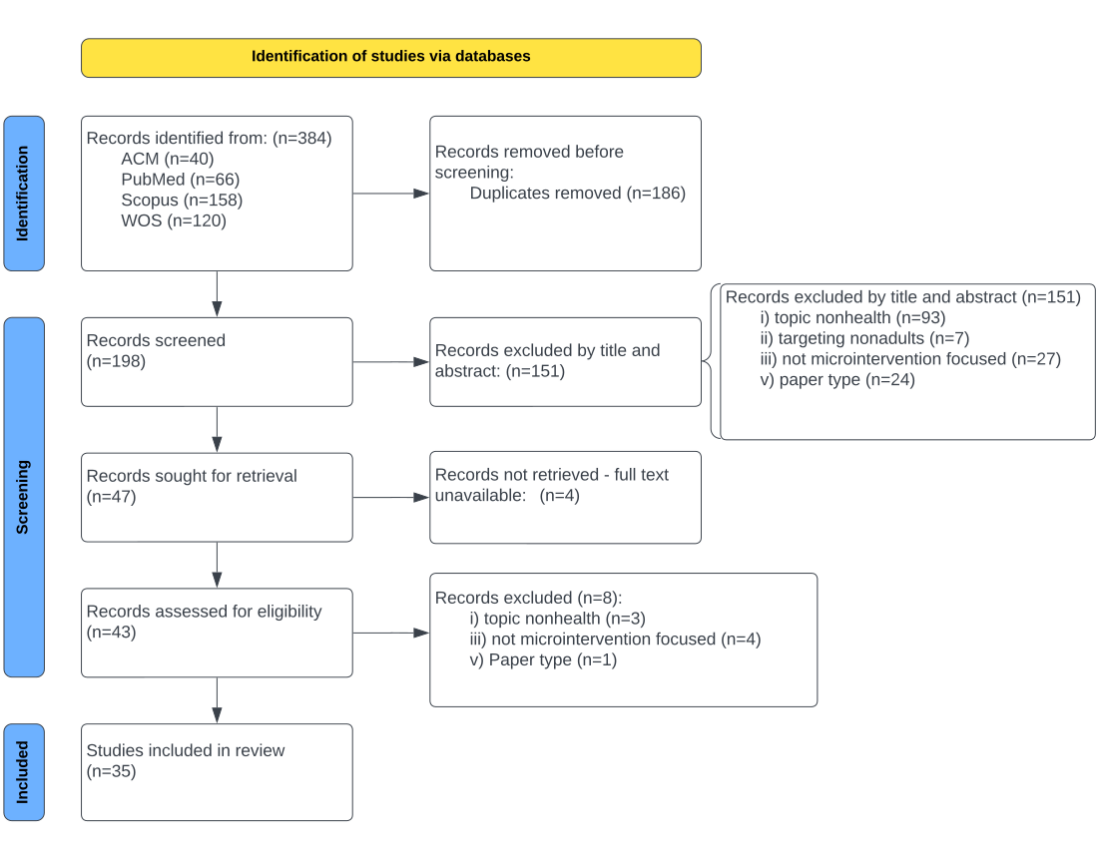
PRISMA (Preferred Reporting Items for Systematic Reviews and Meta-Analyses) flow diagram for June 10, showing the study’s inclusion process. ACM: Association for Computing Machinery Digital Library; WOS: Web of Science.

The PRISMA diagram [52] of the selection process is shown in Figure 1. Tables 1 and 2 provide an overview and breakdown of the included literature. Of the 384 retrieved records, 198 records were screened after removing duplicates. Following title or abstract screening, 151 records were excluded, leaving 47 for full-text review; of these, 4 could not be retrieved, and 8 were excluded based on full-text screening.

**Table 1:** Overview of studies included in the review, ordered by microintervention target and year of publication.

Author	Year	Paper type	Microintervention targets	Microintervention users	Participants (n)	Days	Outcome	Digital (platform)
Tong et al [54]	2023	RT^a^ (4 groups)	Stress	Adults	462	28	Positive	Yes (web-based)
Johnson et al [55]	2022	RT (3 groups)	Stress	Adults	1050	1	Positive	Yes (web-based)
Howe et al [56]	2022	Pilot (3 groups)	Stress	Information workers	86	28	Positive	Yes (desktop and smartphone)
de Witte et al [57]	2022	Development	Stress	All	0	0	N/A^b^	No
Gummidela et al [58]	2021	Mixed experiments	Shallow breathing (stress and anxiety)	Adults	66	1	Positive	Yes (smartphone)
Peeters et al [59]	2020	Pilot (3 groups)	Stress (work-family conflict)	Working mothers	360	7-28	Partly positive	Yes (web-based and smartphone)
Clarke et al [60]	2017	Design	Stress	Adults	0	0	Positive	Yes (smartphone)
Paredes et al [15]	2014	Pilot (4 groups)	Stress	All (adults)	95	28	Positive	Yes (smartphone)
Dainer-Best and Rubin [61]	2024	RT (6 groups)	Mood and anxiety	Adults	350	1-7	Negative	Yes (web-based)
Bunge et al [17]	2023	RT (3 groups)	Mood	Adults	838	7	Positive	Yes (web-based)
Frank et al [20]	2022	RT (2 groups)	Depression, mood, and anxiety	Adults	135	112	Positive	Yes (smartphone)
Kim et al [16]	2022	RT (2 groups)	Mood and motivation	Adults	838	1	Positive	Yes (web-based)
Everitt et al [62]	2021	RT (4 groups)	Mood	All (adults)	235	21-51	Positive	Yes (smartphone)
Meinlschmidt et al [63]	2020	Pilot (2 groups)	Mood	Adults (men)	31	13	Positive	Yes (smartphone)
Meinlschmidt et al [48]	2016	Pilot (2 groups)	QoL^c^ (mood)	Adults (men)	31	13	Positive	Yes (smartphone)
Matthews et al [19]	2014	Pilot	Mood and well-being	Bipolar disorder	3	21-28	Positive	Yes (smartphone)
Davies et al [64]	2024	Design	Body satisfaction	Young women	212	1	Positive	Yes (web-based)
Herriman et al [65]	2024	Pilot	Body satisfaction	Adults	112	1-7	Positive	Yes (smartphone)
Nemesure et al [66]	2023	Feasibility	Body dissatisfaction	Adults	525	1	Positive	Yes (web-based)
Fardouly et al [67]	2023	RT (3 groups)	Body dissatisfaction	Young women	221	14-42	Positive	Yes (web-based)
Fraser et al [68]	2022	RT (3 groups)	Body dissatisfaction	Young women	176	1	Positive	Yes (web-based)
Gobin et al [69]	2022	RT (2 groups)	Body dissatisfaction	Young women	260	1	Positive	Yes
Fuller-Tyszkiewicz et al [14]	2019	RT (2 groups)	Body satisfaction	Women	247	21	Positive	Yes (smartphone and web-based)
Persson et al [70]	2024	RT (3 groups)	Multifaceted^d^	Adults	208	21	Negative	Yes (smartphone)
Xu et al [71]	2024	Case study	Multifaceted^d^	N/A	4	1	Positive	No
Pascoe et al [40]	2022	Feasibility	Multifaceted^d^	Adults	255	196	Negative	Yes (web-based)
Sun et al [72]	2024	RT (2 groups)	Hypertension (blood pressure and healthy behaviors)	Adults	68	84	Positive	Yes (web-based)
Persson et al [39]	2023	Feasibility	Physical activity and healthy diet motivation	Adults	14	14	Negative	Yes (smartphone)
Wahl et al [73]	2022	Feasibility	Compulsive handwashing	OCD^e^	21	1	Feasible	Yes (smartwatch)
Vandesande et al [38]	2022	Pilot	Parent self-efficacy	Parents of children with disabilities	16	21	Partly positive	Yes (web-based)
Malouff and Johnson [74]	2020	RT (2 groups)	Unpleasant dreams	Adults	126	1	Positive	Yes (web-based)
Van Cappellen et al [49]	2020	RT (4 groups)	Enjoyment and motivation to engage in meditation	Adults (midlife)	240	21	Positive	Partly (Ipod)
Conroy et al [18]	2019	Feasibility	Promote PA^f^ and reduce sedentary time	Adults	11	112	Positive	Yes (phone)
Fink-Lamotte et al [75]	2023	RT (3 groups)	Social anxiety and shame	Adults	115	1	Positive	Yes (web-based)
Kivity and Huppert [76]	2016	Diary study (3 groups)	Social anxiety	Adults (high social anxiety)	124	5-9	Positive	Parly (web-based)

^a^RT: randomized trial.

^b^Not applicable.

^c^QoL: quality of life.

^d^Interventions with several targets, for example, stress and physical activity.

^e^OCD: obsessive-compulsive disorder.

^f^PA: physical activity.

**Table 2:** Catalog of microinterventions, event types, the number of events, and resources used by the included studies.

Author	Microinterventions	Event types	Events	Resources
Tong et al [54]	Positive psychology, cognitive behavioral, meta-cognitive, and somatic	Single, repetition, variations, and adaptive	160	Text, URL
Johnson et al [55]	Relaxation, response modulation, positive experiences, and resource buffers	Single	17	Video, text
Howe et al [56]	“get my mind off work,” “feel calm and present,” “think through my stress”	Variations, repetitions	3	Videos, chatbot conversation, and activity suggestion
de Witte et al [57]	Music therapy	Variations	2	Therapist facilitation
Gummidela et al [58]	Game biofeedback, visual feedback, game with pacing, pacing	Repetitions	4	Game, visual, or audio feedback
Peeters et al [59]	“Use your resources” and “Count your blessings”	Repetitions	2	Email, text
Clarke et al [60]	Positive psychology, cognitive behavioral, meta-cognitive, and somatic	Repetitions	18	Apps
Paredes et al [15]	Positive psychology, cognitive behavioral, meta-cognitive, and somatic	Repetitions, single	16	Text, URL to web apps
Dainer-Best and Rubin [61]	Psychoeducation, 5-minute behavioral video, brief behavioral game, brief neutral game, video + behavioral game, video + neutral game	Single	5	Game, audio, video, text, exercises, feedback
Bunge et al [17]	Behavioral activation exercise, reminders, psychoeducation	Single, variations, and repetitions	2	Text, exercise
Frank et al [20]	Personalized messages, psychoeducation	Variations, repetitions	12+	Text, imagery
Kim et al [16]	Behavioral activation (scheduling exercise)	Single	1	Text, exercise
Everitt et al [62]	Mindfulness exercise, relaxation exercise	Repetition	4	Audio exercise
Meinlschmidt et al [63]	Viscerosensory attention, emotional imagery, facial expression, contemplative repetition	Single, repetition	4	Short video clip
Meinlschmidt et al [48]	Viscerosensory attention, emotional imagery, facial expression, and contemplative repetition	Repetitions	4	Text, videos, audio
Matthews et al [19]	Visualized digest	Repetitions	1+	Visualization
Davies et al [64]	Appearance-related self-compassion, humorous or factual messages	Single	15	Text, imagery
Herriman et al [65]	Psychoeducation, thought challenge, and written interactive task	Single, adaptive	1	Video, exercise
Nemesure et al [66]	Minicourse (questions, feedback, exercises, psychoeducation)	Single	1	Chatbot, text, exercises, feedback
Fardouly et al [67]	Social media posts (eg, body diversity, body acceptance, and kindness)	Single, variations, and repetitions	42	Text, imagery
Fraser et al [68]	Gratitude meditation, mindfulness meditation	Single	2	Audio guide
Gobin et al [69]	Self-compassion	Single	1	Written task
Fuller-Tyszkiewicz et al [14]	Gratitude task, breathing, and relaxation	Variations, repetitions	11	Videos (education and exercises)
Persson et al [70]	Self-guided generation task, audio-based projection, image-based projection	Variation, repetitions	6	Audio, text, imagery, exercises
Xu et al [71]	Movement-based interventions, for example, dance and calligraphy	Single	4	Phone call
Pascoe et al [40]	Psychoeducation, exercises, general education	Variations, repetitions	22+	Web-based (videoconference)
Sun et al [72]	Education, persuasion, incentivization, training, environmental restructuring, enablement	Single, repetition, variations, and adaptive	12+	Video, text, exercises, imagery
Persson et al [39]	Self-guided generation task, audio-based projection, Image-based projection	Variation, repetitions	3	Audio, text, imagery, exercises
Wahl et al [73]	N/A^a^	N/A	0	N/A
Vandesande et al [38]	Psychoeducation	Sequence	4	Video series
Malouff and Johnson [74]	Vivid recall of positive events	Single	1	Text
Van Cappellen et al [49]	Loving-kindness meditation, or mindfulness, and educational passage	Single, sequence, and repetitions	4	Text (educational), audio (exercise)
Conroy et al [18]	Motivational, educational, or action messages	Variations	456	Text
Fink-Lamotte et al [75]	Self-compassion, cognitive reappraisal, and a control intervention	Single	3	Audio
Kivity and Huppert [76]	Reappraisal, reminders	Repetitions	2	Reappraisal training (in-person), text

^a^Not applicable.

The majority (28/35, 80%) of the studies included were published after 2020, highlighting an increasing interest in microinterventions. Little under half of the included studies (16/35, 46%) describe forms of randomized trials, with the other half (19/35, 54%) primarily pilot, developmental, or feasibility studies. A common trend among studies (13/35, 37%) is the focus on in-moment effects, limiting study duration: often less than one day. More long-term studies (18/35, 51%) generally lasted 7-31 days, except for 4 (11%) studies lasting 84-196 days [18,20,40]. The number of participants varied significantly across studies, between 0 and 1050 participants. In the study with 0 participants, microinterventions were based on existing literature and were reviewed by a group of experts rather than potential end users [57]. The events presented, that is, the unique attempts at positive momentary change, were generally relatively low, ranging between 1 and 18 (30/35, 86%), with notable exceptions of 0 and 456 events. Although the paper by Wahl et al [73] does not present any events or microinterventions, it is included in the review due to its focus on when to deploy these (JIT recognition, ie, a component of a microintervention) rather than which microintervention or event should be used. The paper by Baumel et al [12] is not formally included in the review as it does not implement any microinterventions or components (inclusion criterion 3) but rather theorizes on the structure of microinterventions. The constructs and definitions presented by the paper were, however, included in the CIS process as they were foundational for a number of the included papers [56,61,64,68,72]; no similar papers have been found nor excluded as part of the review process.

### The D-MIST Framework

#### Overview

This section presents the D-MIST framework, including its components and their rationale. The framework and its components are described and exemplified using the included literature on microinterventions (listed in Table 1), with a consolidated description of their components sorted by key concepts. The D-MIST framework is a synthesis of the reviewed literature, including Fuller-Tyszkiewicz et al’s [14] definition of microinterventions, Baumel et al’s [12] conceptualization of microintervention care, and other uses seen in the literature. The framework aims to unify these—at times disparate—concepts into a single framework with concrete evidence-based definitions.

**Figure 2:**
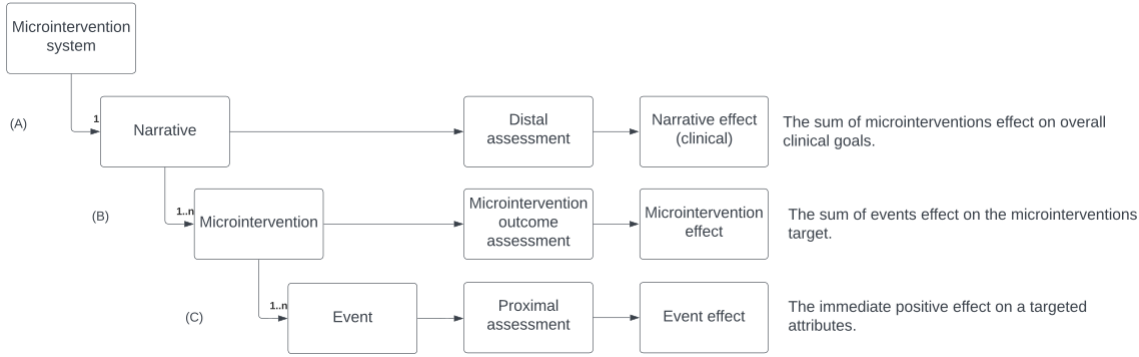
The Design Framework for Microintervention Software Technology (D-MIST) framework of microintervention systems with three levels: (A) narrative, (B) microintervention, and (C) event. At each level, there are different effects. The narrative focuses on the distal perspective and is encountered by the user through a number of microinterventions, each of which has its own effect. Each microintervention contains one or more events that focus on the immediate or proximal perspective.

The D-MIST framework is illustrated in Figure 2 with the constituent concepts defined in Table 3. The framework defines a microintervention system on three levels: (1) the overall narrative level, (2) the microintervention level, and (3) the event level. The overall effects are measured through the narrative effect, which is the sum of its constituent microintervention effects. The microintervention effect is, in turn, the sum of the event effects.

The narrative is the consolidated experience of engaging with multiple microinterventions and their events over time, aiming to accomplish an overall narrative goal, for example, a clinical goal. The microintervention is a highly focused intervention consisting of one or more in-moment events of a given type, each attempting a positive effect. In this way, microinterventions can be seen as self-contained programs of events aiming to facilitate small but meaningful effects, in support of the microintervention effect and, in turn, the narrative effect. The event is the delivery of a resource, for example, an exercise or educational video, aiming for an immediate positive effect. An event may, depending on its type, do different things: present variations of an exercise to maintain engagement over time; be designed for repeated use, or be part of a sequence of logically ordered events.

**Table 3:** Definitions of microintervention systems and their core components.

Components	Definitions
Microintervention system	The microintervention system, through its design and interaction model, aims to facilitate (1) the creation and maintenance of a narrative, (2) by serving relevant microinterventions, and (3) through recognition of the user’s situation, context, and needs.
Interaction model	The interaction model formalizes how the aims of the system are achieved through the user’s interactions with the system (eg, JIT^a^ and on-demand) and other actors (eg, health coaches and other users).
Narrative	A narrative aims to address a clinical goal by creating a meaningful, connected story through its microinterventions and the events as experienced by the user.
Narrative thread	The narrative thread is the list of experienced microinterventions through their corresponding events, where each step is determined by the users’ circumstances and the best-fitting conceptual model.
Microintervention	A microintervention is a highly focused type of therapy relying on one or more events of a given type, each attempting some momentary change.
Conceptual models	Conceptual models describe the conditions for when and where a microintervention is relevant and can be used as part of a narrative.
Event	An event provides resources as required by its intended types and therapeutic groups, with an immediate positive effect on target attributes.
Decision rules	Decision rules determine which events should be triggered, the intensity of the event, and the conditions under which they should be triggered (eg, contextual factors, time of day, or user’s state).
Type	The event type refers to the way the event is used. Events can be classified as single-use, repeated, varied, sequential, or adaptive.
Resource	Each resource provides a piece of content (eg, a video, exercise, or call to action) in various formats, aiming to facilitate an immediate positive effect on target attributes.

^a^JIT: just-in-time.

A microintervention system may, for example, consist of a smartphone app with the intended effect (narrative goal) of improving sleep. The narrative might then consist of a sequence of microinterventions with their own intended effects (microintervention goals; eg, avoiding nightly screen time, mindfulness, and stress reduction) which through a number of events with their intended effects (event goals; eg, a video on the effects of nightly screen time, calls to action, and relaxation exercises) deliver various educational video and exercise resources.

A microintervention system aims to create and maintain a narrative through recognition of a user’s situation and needs. In practice, these aims are accomplished through the interaction model formalizing how the users interact with the system (eg, JIT and on-demand) and other actors (eg, other users and health coaches). An on-demand system allows users to choose which microinterventions and events to engage with and when to do so. Other system designs may have a general practitioner or the system itself influence which microinterventions users engage with, perhaps through automated recognition of the user’s situation, context, and needs.

The selection of microinterventions is supported by conceptual models detailing conditions for when a microintervention is relevant. In JIT- or JITAI-like systems, these conditions are explicit, detailed, and operational. They may alternatively be implicit from a design perspective when users are allowed to choose their microinterventions.

Once a microintervention is chosen, the constituent events are typically deployed either randomly or through more specific decision rules such as time of day or time since last event [56]. These decision rules may also be explicit or implicit. In on-demand systems, users may consume events at their own pace [22] or all at once, while JIT-type systems may have explicit operational rules allowing precise contextual deployment.

Applying the D-MIST framework to the system presented by Frank et al [20] (Multimedia Appendix 2 [20,40]), we see the system as a smartphone app enabling feature monitoring, self-assessment, psychoeducation, and delivery of personalized microinterventions with the narrative goal of improving mood. In practice, this is accomplished through an interaction model consisting of the system, clinician, and end user. The system facilitates data collection, allowing clinicians to identify needs and suggest relevant microinterventions, which users receive through the smartphone app. Users can then consume or reject suggested microinterventions, forming a collaboratively tailored narrative thread. While the study [20] does not explicitly report on provided narratives or conceptual models, examples suggest microinterventions did not always target mood directly: for instance, one microintervention goal was to improve sleeping habits, with the event goal being to reduce nightly exposure to smartphone light.

Looking at other examples from the literature, we could imagine narratives targeting mood to include interventions such as behavioral activation [17] and relaxation exercises [62]. Moreover, based on the work of Malouff and Johnson [74], events aiming to reduce unpleasant dreams could also improve sleeping habits.

However, as noted in the introduction, it is difficult to evaluate which leveraged microinterventions and events made a difference if these are treated as a part of a “black box.” Some users may, for instance, not benefit from improved sleeping habits, making these components irrelevant in such cases. Instead, using D-MIST’s levels to describe microintervention systems not only allows designers a means of reporting on individual narrative threads but also allows descriptions of each used microintervention, their events, and when these may be used in a narrative. Multimedia Appendices 2 [20,40] and 3 map a number of other component examples. In the following subsections, we will provide a narrative overview of the literature and rationalize the framework, followed by a short overview of efficacy and user experience insights from the included literature.

#### Microintervention System

Among the systems included in our literature review (Table 1), most are fully digital (31/35, 89%), with smartphones (14/35, 40%) and web-based apps (18/35, 51%) being the most used platforms. Howe et al [56] describe three overall ways of interacting with microinterventions: JIT, prescheduling, and on-demand. JIT refers to the system choosing the intervention time [62], the intervention [15], or both [56]. Prescheduling systems allow users to choose interventions ahead of time or to choose an opportune time for engaging with an intervention [56]. On-demand is when users can choose when to engage with interventions freely from available microinterventions [14]. These patterns are not mutually exclusive, as illustrated by Howe et al’s [56] study, where on-demand features were available to both the JIT and prescheduled study conditions. It can be argued that Meinlschmidt et al [48] leverage elements of both prescheduling, as engagement is expected daily, and on-demand, as users can choose their intervention and specific time during the day. Paredes et al [15] let users choose when to request a microintervention while using JIT elements to recommend an intervention relevant to the user’s context.

**Figure 3:**
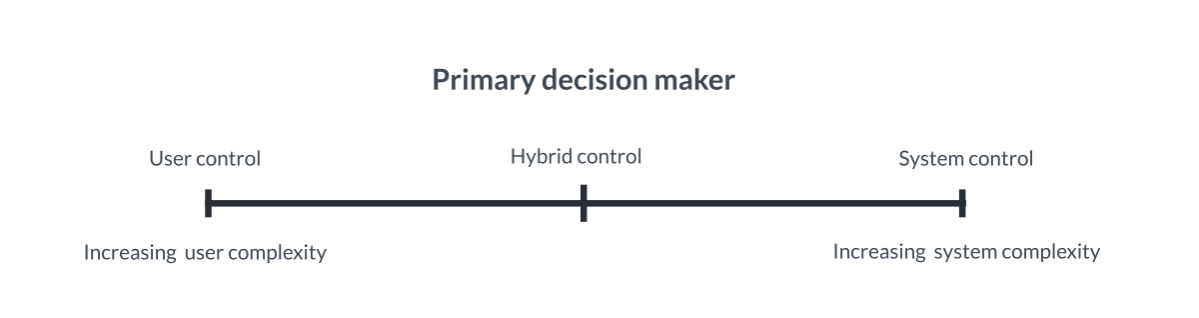
Decision-making continuum for microintervention systems.

Many microintervention systems rely on users initiating when to engage [14,15,40,48,56]. This may be based on their own sense of state and context [76], or when possible, specified external factors [62]. Most systems make use of hybrid approaches where both systems and end users are involved in decision-making [15,56]. However, there are also examples of predominantly user-centered [48,49,63] and system-centered decision-making [18,62]. As illustrated in Figure 3, this decision-making relationship can be seen as a continuum from predominantly user to system-controlled, with most systems falling into a hybrid category with various blends of the two, covering the levels of automation previously conceptualized [77]. Importantly, this continuum may itself be subject to user preference; that is, users may have different preferences for the balance between automation and agency [56].

The system or user decision-making process can, however, also be more complex, involving other users [15], clinicians [20], or be context-aware [19]. Narratives can be provided by the system through decisions made by a clinician [20,57], digital apps [18,60], the end users [58,59,76], and combinations of these actors [15,48,56].

In practice, users’ needs in such systems can be recognized in several ways, such as by users through subjective self-assessment [14,48,56], supported self-assessment, for example, visualization [19], clinicians reviewing data [20], or objective system assessment through recommendation techniques [54,60]. Machine learning has, for example, been used to trigger interventions in response to the user’s actions, as seen possible with the identification of compulsory hand washing [73] and workplace stress [56]. However, it may also be used to identify the most effective intervention for a specific user [15]. Clinicians may likewise act in this role, recognizing a user’s state and context and suggesting relevant microinterventions [20,57].

The D-MIST framework defines the microintervention system as delivering a narrative through its design and interaction model. We base the aims of the microintervention system on Baumel et al’s [12] description of a hub: an entity that (1) recognizes an individual’s state and context, (2) recommends relevant interventions, and (3) helps create or maintain the narrative derived from each intervention as part of the larger therapeutic process. However, we also observe notable limitations with this description. First, it does not explicitly and adequately describe the roles of the system [48,56] delivering the narrative experience. Second, its emphasis is on a single “entity” [12] primarily fulfilling these aims rather than multiple actors collaboratively [19,48,56]. JIT has, for instance, been leveraged to recommend when participants should engage in a microintervention in line with point (1); but with users determining which intervention to engage with in line with point (2) [56], highlighting how multiple actors may collaboratively fulfill the aims outlined by Baumel et al [12]. Third, interactions between actors (user, system, and others) as observed in the literature [15,19] are not explicitly modeled. As highlighted above, microintervention systems use different ways of interacting with microinterventions, levels of automation, user roles, and actors. Despite these differences, the overall aim of the system remains in line with Baumel et al’s [12] definition. Given the many different ways these aims can be accomplished through the system, its actors, and interactions, we define the role of the microintervention system as:

The microintervention system, through its design and interaction model, aims to facilitate (1) the creation and maintenance of a narrative, (2) by serving relevant microinterventions, and (3) through recognition of the user’s situation, context, and needs.

We subsequently define the interaction model as:

The interaction model formalizes how the aims of the system are achieved through the users’ interactions with the system (eg, JIT and on-demand) and other actors (eg, health coaches and other users).

Not only do these definitions help outline the system’s role in delivering microintervention narratives, but they also formalize how narratives are formed through interactions between user, system, and other actors. A well-described interaction model also allows us to more easily compare the advantages and disadvantages of different approaches. We note that microintervention care has been defined by Baumel et al [12] to refer to the abstract therapeutic process of using microinventions as building blocks; we have opted for a narrower definition here, centered around the facilitating microintervention system encompassing identified building blocks.

In the example using Frank et al’s [20] system, we note how users and clinicians collaboratively create a narrative, based on the clinicians’ understanding of user needs. Compared to interaction models where users themselves freely choose between microinterventions such as those by Howe et al [56] and Meinlschmidt et al [63], the approach by Frank et al [20], where clinicians provide microinterventions, may result in higher effectiveness but at the potential cost of user agency and self-exploration. Similarly, while JIT-based interaction models may recommend interventions at seemingly optimal moments, these may nevertheless appear contextually inappropriate for users [62,70].

Having looked at the role of microintervention systems and how narratives emerge from interactions, it is worth returning for a moment to the rationale for Figure 2. The system, through its interactions, provides a consolidated narrative experience over time, in some cases initially consisting of one microintervention with more added over time. The system by Frank et al [20], for instance, initially consists of an educational microintervention with additional microinterventions and events provided over time depending on collected data. Pascoe et al [40] similarly present several microinterventions targeting, for example, stress, physical activity, and sleep in support of the overall narrative well-being goal, with one example being a mindfulness stress microintervention. However, each microintervention can also have more than one event [12,14]. Fuller-Tyszkiewicz et al’s [14] mindfulness intervention, for example, consists of 9 events. However, we will describe the rationale for these components further in the following subsections.

#### Narratives

Narratives, where multiple microinterventions are used in combination, occur in more than half the literature (20/35, 57%), with 6 of these papers considering broader narrative implications. Narratives do not occur in 15 (43%) studies as these test microinterventions in isolation.

Paredes et al [15] create a narrative by having users request a microintervention for stress, with the system suggesting the most effective one based on user traits and the circumstances. Conroy et al [18] randomized the time at which interventions arrive and selected a random message from three categories promoting physical activity. Clarke et al [60] describe how the system chooses between 18 interventions initially at random and later based on how effective individual microinterventions were at alleviating stress, with the system aiming to provide personalized treatment, that is, an individually effective narrative. Tong et al [54] tested three ways of creating narratives through their study conditions: self-chosen interventions, randomized interventions, and recommended interventions, concluding that self-chosen and recommended narratives were equally effective at stress reduction. Meinlschmidt et al [48,63] had users themselves be responsible for determining the narrative by choosing between 4 microinterventions, whereas Everitt et al [62] had users choose between interventions for improving mood, but also had the system provide JIT prompts for engagement.

Fuller-Tyszkiewicz et al [14] present a narrative of 11 ordered events which can be viewed as two microinterventions, mindfulness and gratitude, but note that participants were free to consume or repeat these and their events in any order they liked. Fuller-Tyszkiewicz et al [14] also suggest that digital motivational content may increase motivation to engage in future microinterventions, implying there may be benefits from combining different interventions in terms of both attrition and overall effect [14,55]. Two studies investigated this by considering the narrative implications of microinterventions, increasing the effectiveness of subsequent microinterventions, for example, by reducing stress-related barriers to concentration and learning [57]. Other work suggests this extends to depression as well, which may present a significant barrier to engagement, for example, in diabetes [78]. The other narrative consideration is the introduction of educational interventions, enhancing the enjoyment of, and motivation to engage in, subsequent microinterventions [49]. Results suggest one microintervention successfully amplified the effects of another: that is, the effect of a loving-kindness meditation intervention was amplified by priming through a prior microintervention [49].

Despite most studies using a limited number of microinterventions and events, these still form “accounts of connected events,” that is, a narrative through the systems design and interaction model. Given that events aim to have an immediate effect on targeted attributes measured through proximal assessments [12], it stands to reason that a microintervention’s effect is the synergized sum of event effects over time. Consequently, the effects of a narrative can be viewed as the synergized sum of its microinterventions with the events experienced by the end user. The narrative aim may be different from that of the individual microinterventions and their events, as seen, for example, in the work of Gummidela et al [58], where shallow breathing (an event effect) is targeted as a proxy for stress (a microintervention effect). Sun et al show support for this, as improvement of microintervention goals, for example, physical activity and medication adherence, results in improvements to the narrative goals, that is, blood pressure [72]. Consequently, we see three effect scopes and assessments that can be used: proximal assessment, looking at the individual event effects; microintervention outcome assessment, gauging the overall microintervention effect; and distal assessment, gauging the narrative effect. In type 2 diabetes, for example, the overall aim (distal effect) might be to reduce glycated hemoglobin [79], with individual microinterventions aiming to improve self-management, through events aiming at reducing discounting of the future [80,81]. Thus, the narrative structure is responsible for creating a meaningful experience toward achieving the distal treatment goal of the system, trying to synergize the effects of different microinterventions. We thus define the narrative and the narrative thread as:

A narrative aims to address a clinical goal by creating a meaningful, connected story through its microinterventions and the events as experienced by the user.

The narrative thread is the list of experienced microinterventions through their corresponding events, where each step is determined by the users’ circumstances and the best-fitting conceptual model.

This definition stands in contrast to Baumel et al [12], which describes the narrative as the linking agent between interventions rather than referring to the consolidated experience. However, by emphasizing the narrative as the consolidated experience, D-MIST aims to promote explicit consideration for how microinterventions are used and combined over time to achieve distal outcomes. Narratives should aim to engage users through the deployment of meaningful, connected, and goal-relevant microinterventions.

Low-intensity microinterventions could, for example, be used to promote initial engagement before transitioning to higher effort but more effective interventions [56]. Other early microinterventions could reduce barriers to engagement, such as discounting of the future [70] and stress [57], and later microinterventions may attempt to address rising needs for novelty [15]. Additionally, some users may not need certain microinterventions or may have a preference against some types of microinterventions [70], making others more relevant in a narrative.

The sparse narrative insights presented throughout the reviewed literature may hint that narrative considerations could be key to increasing perceived usefulness [56], reducing attrition [14], increasing or supporting the effectiveness of other microinterventions [49], creating personally relevant experiences [30], and addressing changing needs over time, for example, a need for novelty [56]. The narrative thus presents significant opportunities for personalization in addressing needs and should aim to create a coherent experience over time in pursuit of the distal narrative goals.

#### Microinterventions

The majority of the microinterventions included in our review (Table 1) focus on either reducing stress (8/35, 23%), improving mood (8/35, 23%), or body dissatisfaction (7/35, 20%). The remaining studies (12/35, 34%) have various targets such as self-efficacy of parents [38], reduction of unpleasant dreams [74], compulsive hand washing [73], promotion of physical activity [18], hypertension [72], or have multiple targets [40,70,71].

Digital interventions affecting stress include four overall therapy groups (or categories of microinterventions) [15], namely: (1) Positive psychology, (2) Cognitive-behavioral, (3) Meta-cognitive, and (4) Somatic [15,54,60], that is, Paredes et al [15] use these groups along with the nature of the intervention (individual or social) to classify their microinterventions consisting of a prompt and a link to the intervention resources. Another, more indirect approach to stress consists of targeting shallow breathing as a proxy for stress and anxiety [58]. Other proposed categories for stress reduction include relaxation, response modulation, positive experiences, and resource buffers [55].

Two exceptions to the norm of digital interventions are music therapy [57] and dance movement therapy [71]. In music therapy, a clinician uses musical instruments with the patient to reduce stress by attunement through music [57]. In dance movement therapy, a clinician suggests personalized physical exercises over the phone to, for example, alleviate stress. While these interventions are not facilitated through digital means, they are similar to the social interventions described by Paredes et al [15], which rely on the inclusion of other actors.

A common type of microintervention is the delivery of exercises such as mindfulness and relaxation [62] or suggesting activities like various forms of meditation [49,68] and behavioral activation [16,17]. We see this reflected in the studies looking at body satisfaction where meditation is used [68], in addition to self-compassion [69], themed social media posts [64,67], and exercises such as breathing, gratitude, and relaxation [14]. The first and last of these are used to elicit mood change as well [62]. Other implementations include the reduction of social anxiety through reappraisal [75,76] and increasing physical activity through educational, motivational, and actionable messages [18]. Microinterventions also leverage more passive approaches, such as a visual digest of mood [19], allowing users to react when they feel it necessary.

One issue created by the definitions of a microintervention so far is the ambiguity of what can be considered a single microintervention and an event. We observe, in line with Fuller-Tyszkiewicz et al’s [14] description of microinterventions, that the events may be single-use, designed for repetition, or offer content variations to maintain user interest. However, a microintervention may also consist of other event types: for example, sequential events. We base this on the results of our review, specifically that some microinterventions may have prerequisites [39,70] or be sequential [14] with, for example, Vandesande et al [38] using sequential videos to deliver psychoeducation. While it could be argued that each video in this example in itself is “one” single-event microintervention with an effect, knowledge from one video may be necessary to understand another video’s content. Thus, subsequent events are not isolated and cannot be considered a series of single-use microinterventions that are part of a narrative, but should rather be considered as one microintervention consisting of several events to be consumed in order. A number of works nevertheless use single-event microinterventions [16,66], for instance, a minicourse positively impacting body image [66]. These may be reminiscent of prior works on “single-session interventions” [82]. Consequently, we define a microintervention as:

A microintervention is a highly focused type of therapy relying on one or more events of a given type, each attempting some momentary change.

As with previous definitions of microinterventions, this still creates some ambiguity as to what can and cannot be considered a single microintervention; however, we believe this ambiguity is a question of design, that is, several different microinterventions may target the same symptom or attributes but be designed in such a way that they rely on different numbers of events and types of resources. Similarly, each design may be subject to different conceptual models for when to make narrative use of each microintervention based on user needs, preferences, and other factors.

Returning to the previous example on sleeping habits, a focused microintervention may attempt to reduce unpleasant dreams [74] through repeated daily reflection events; another may aim to motivate users to wake up at the same time every day by suggesting a variety of behaviors such as going for a walk or meeting friends for coffee [20]. A microintervention could also aim to reduce factors affecting sleep, such as late-night screen time [20], through single-use events or a sequence of educational events.

#### Events

**Figure 4:**
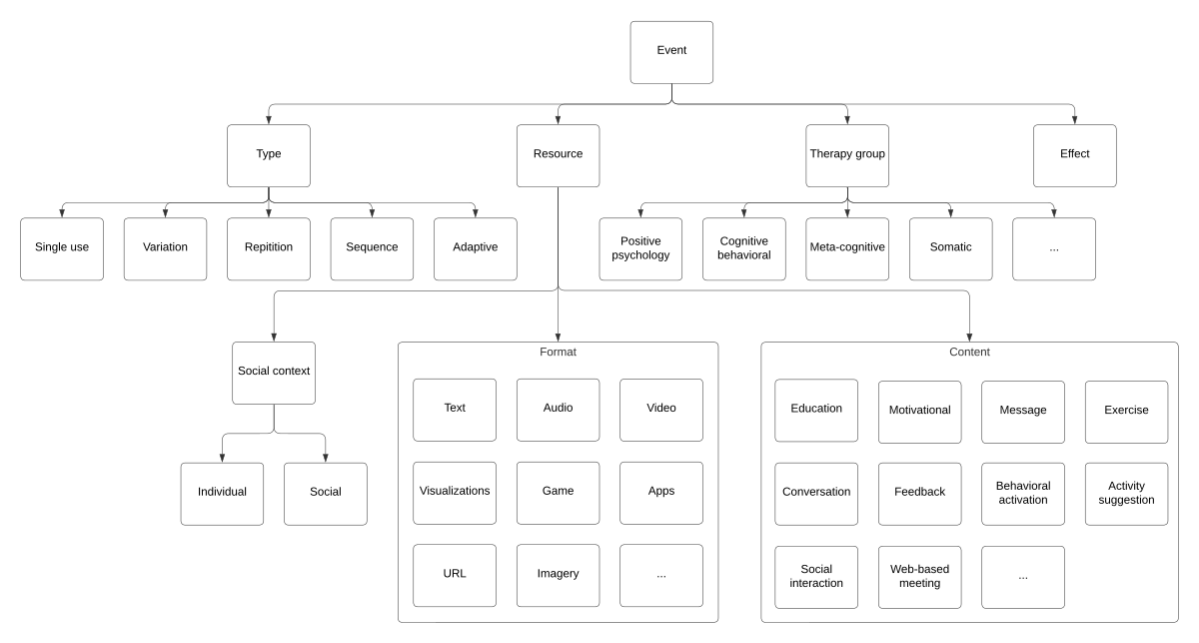
Summary breakdown of events as seen throughout the included literature.

The number of events and how these are used in individual works differ significantly; an overview of event components can be seen in Figure 4.

Conroy et al [18], for instance, include 456 unique events, while the work by Tong et al [54] contains 160. While all these types of events can be classified as “single” events, they could also be seen as “variations” to maintain engagement over time [14]. A number of studies further test single-event microinterventions, including Kim et al’s [16] behavioral activation exercise affecting mood and Gobin et al’s [69] self-compassion exercise affecting body dissatisfaction. While these two examples only used each event once, these could be used repeatedly, as seen in other works [48,56,59,62,63]. The exercises presented by Paredes et al [15], meanwhile, present variation in that the resource content provided can differ between uses.

Events may also form logical sequences [38,39,49]. Vandesande et al [38], for example, include the use of psychoeducation to build up self-efficacy knowledge through several videos. Van Cappellen et al [49] use one event to increase the enjoyment and continued practice of meditation events, that is, a form of priming, while Persson et al [39] use one event as a prerequisite for others.

Events can also be adaptive [54,65], serving resources that match users’ momentary abilities; a microintervention serving short physical activity exercises may, for example, attempt to adapt the resource to match users’ exercise level [83,84]. Events may adapt the resource itself to the context or its dose [11] or provide “variations” to maintain interest and engagement [14]. Another somewhat extreme example of an adaptive event is prompting users to “Take a moment and do whatever comes to mind to reduce your stress” (Tong et al [54]).

The length of the events presented also varies. The messages used by Conroy et al [18] can be consumed within a matter of seconds, while Johnson et al [55] tested both short 2-minute and one 20-minute microintervention. Despite educational events being between 10 and 19 minutes [38,40], most events last a few minutes [14,15,20,48,54,69,74], indicating that a few minutes is perceived to match users’ capacity for engagement [12,55]. Authors such as Tong et al [54] and Paredes et al [15] also emphasize that events should last less than 3 minutes, while Baumel et al [12] simply emphasize that the duration should match users’ capacity more broadly. As explored in previous examples, resources delivered by events provide a multitude of content (eg, psychoeducation and exercises) through various formats (eg, text and video), an overview of which can be seen in Figure 4 and Table 2.

Baumel et al [12] have previously described events as the smallest element of the microintervention, which attempts some in-the-moment change or impact toward intervention targets. This is accomplished through the event’s resource [14] and served according to its type. Events can be classified based on therapeutic group or on whether they can be consumed alone or with others [15]. Furthermore, some events may have prerequisites, interdependencies, or logical order to them and may be adaptive in nature.

Based on the literature, we have identified five overall types of events: single-use events— where a single resource is used exactly once. Repetition events—where a single resource is used repeatedly. Variation events—where variations of a resource are used repeatedly. Sequential events—where resources are order-dependent or in a logical sequence. Adaptive events—where the resource itself or the intensity varies or is tailored to users. Based on these insights, we defined an event as:

An event provides resources as required by its intended types and therapeutic groups, with an immediate positive effect on target attributes.

We define event types as:

The event type refers to the way the event is used. Events can be classified as single-use, repeated, varied, sequential, or adaptive.

We use the term “attribute” rather than “symptom” as events may target not only symptoms but also more abstract targets such as enjoyment or motivation to engage in subsequent interventions. Resources may further be defined as:

Each resource provides a piece of content (eg, a video, exercise, or call to action) in various formats, aiming to facilitate an immediate positive effect on target attributes.

The event presented by Malouff and Johnson [74], for example, used a text-based resource containing instructions for a small exercise done before bed, aiming to reduce unpleasant dreams, which can be used once (or potentially repeatedly). A microintervention aiming to reduce nightly screen time could use resources such as educational videos or JIT text-based warning or reminder notifications to achieve immediate effects [20].

#### Decision Rules, Conceptual Models, and Assessments

In this section, we cover insights on when individual events are deployed through decision rules, when microinterventions are relevant in a narrative through conceptual models, and which assessments are used.

Decision rules are often triggered by combinatory [85] data collection [15,20,56]: system-driven [60], user-driven [62]. Collection occurs both by subjective assessments using questionnaires and objective system-driven data collection, for example, biometrics [56,60]. In mood interventions, we see both collection types used through assessments and the collection of GPS, conversational feature identification, and sleep monitoring [19]. Examples of user-driven data collections include the recording of meals, ecological momentary assessments such as mood check [62], and standardized assessments such as the Multidimensional Mood State Questionnaire [63].

**Figure 5:**
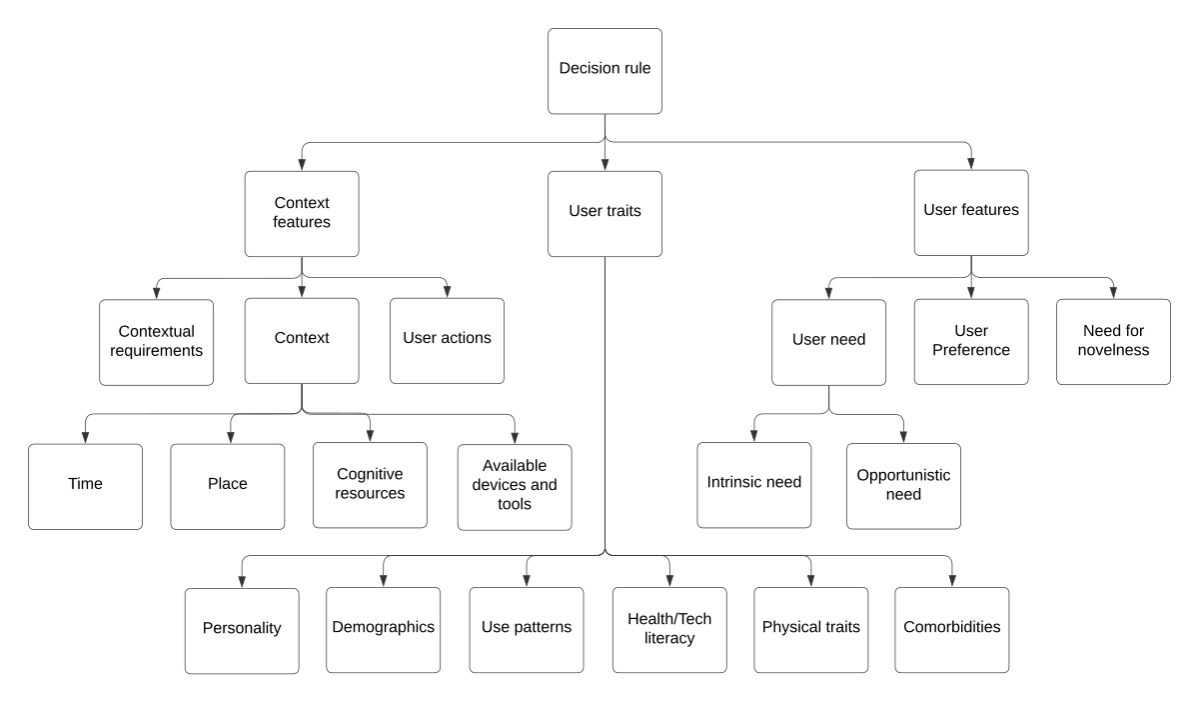
Summary breakdown of microintervention decision rules as seen throughout the included literature.

Howe et al [56] use six decision rules for event deployment, including a computed stress score (based on email volume, calendar saturation, time, facial expression, heart rate, and assessments) and 5 timing-related rules, for example, within working hours and no intervention completed in the past hour. Tong et al [54], meanwhile, use machine learning to suggest the most effective events based on users’ stress score, the number of tabs open, and user settings. Stress detection can also make use of specialized technology such as webcam-based heart rate monitoring [56], smartwatches [60], and a harness chest strap for measuring breathing rate [58]. The “mStress” app, for example, uses three modules: a stress recognition module, a set of stress interventions, and an intervention recommender module based on a Q-Learning algorithm [60]. Other system-driven data collection methods used are activity monitors [18] and smartwatches [60,73]. A summary breakdown of factors considered in decision rules can be seen in Figure 5.

Many decision rules are also presented more implicitly; for example, several studies allow users to choose between interventions or events based on a prior introduction to these [14,48,56]. Howe et al [56], for instance, introduce users to interventions through a home screen showing a title, image, and short textual descriptions of the intervention.

**Figure 6:**
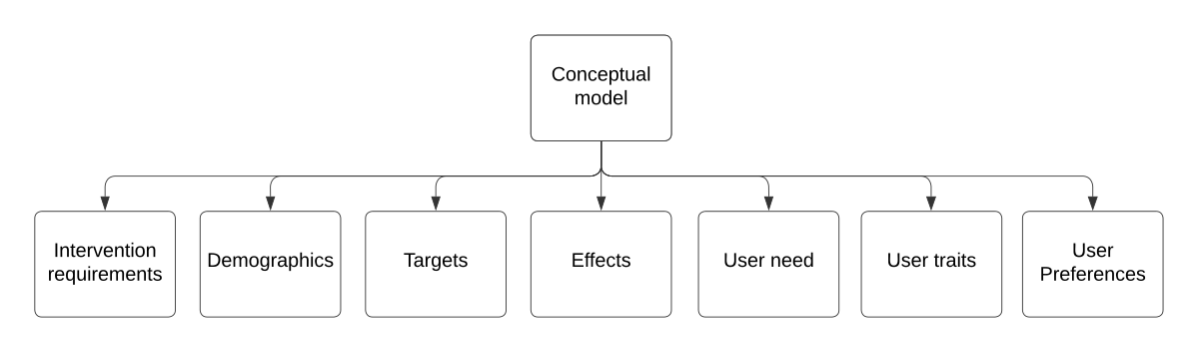
Examples of possible components of conceptual models used to determine intervention relevance to the narrative.

While no studies explicitly address conceptual models for various microinterventions, some can be derived from reported results (Figure 6). We note that many of these share the attributes seen in decision rules. All presented interventions have well-described targets such as stress, mood, or body dissatisfaction, or physical activity motivation [18] or parent self-efficacy [38]. Other details on applicability, such as those exemplified in Figure 6, including demographic targets or user traits, are sparser.

Some microinterventions have multiple purposes; for example, mindfulness has been used to improve both mood [62] and body satisfaction [68], and other meditation exercises have been used to affect sleep, anxiety, and stress [86-88]. Thus, interventions may have multiple targets reflected by different conceptual models.

However, the conceptual models may also be more implicit: Pascoe et al [40], for example, allow users to tailor their own well-being narrative by providing a choice to join a daily session, comprising six microinterventions affecting, for example, stress management, physical activity, or improving sleep, with event content varying between days.

A number of assessment methods have been used to inform both decision rules and conceptual models, such as Patient Health Questionnaire-8 or -9 [15,20,62] and state body image [69]. Nemesure et al [66] used two 5-point scales to determine perceived body image and motivation to improve body image, and Kim et al [16] used a 9-point Likert scale to measure mood and confidence in mood self-improvement.

Generally, we see significant similarities between decision rules and conceptual models. User traits and preferences may both inform whether an intervention should be used, but may likewise affect the timing of interventions, for example, situational appropriateness. Decision rules (often called triggers within the field of software engineering) aim to detail when and where events should be deployed [12]. While relatively few papers explicitly report decision rules, insights can be drawn from both proximal and distal assessments used by these studies. Many assessment methods are therefore interesting not only for their ability to determine in-moment effects and distal outcomes but also to inform conceptual models determining whether a particular type of microintervention could be used in a certain context [12].

Thus, conceptual models determine which of the available microinterventions are being served, with specific timing controlled by the decision rules. Moreover, given that microinterventions can be JITAI in nature [14], we add, in line with Nahum-Shani et al’s [23] definition of JITAI decision rules, that these can also affect the intensity. We thus define decision rules as:

Decision rules determine which events should be triggered, the intensity of the event, and the conditions under which they should be triggered (eg, contextual factors, time of day, or user’s state).

We define conceptual models as:

Conceptual models describe the conditions for when and where a microintervention is relevant and can be used as part of a narrative.

For example, a prerequisite for the deployment of sleep habit microinterventions in a mood narrative is the need for such interventions reflected by a conceptual model, identified through detection of nightly screen time or poor sleep rhythms. However, if users do not have a need for such microinterventions, it is not meaningful to deploy them. Instead, users are better served by other microinterventions.

In the case of events aiming to reduce unpleasant dreams, the only decision rule for deployment relates to whether users are about to head to bed, while JIT screen time events could consider screen activity, time of day, time since last event, and which events are best to deploy, for example, educational, motivational, or calls to action.

#### User Experience and Personalization

Given that much of the potential of microintervention technologies lies in their flexibility and personalization potential, we want to highlight some of the user experience insights found in the literature. User experience and usability were explicitly assessed and reported in some studies (15/35, 43%), with usability, acceptance, and interest rated highly when reported [14,38,48,56,58,60,62]. The study by Howe et al [56] finds that on-demand may have advantages over prescheduled interventions as participants choose to use the on-demand feature when stressed, causing greater reductions. Participants themselves seemed to prefer JIT, despite some indications that prescheduled may be more effective for behavior change. Similarly, another study found that JIT did not have tangible benefits over the non-JIT condition, noting this could be due to contextual inconveniences of interventions [62]. Another study noting similar inconveniences noted this could be due to a lack of user involvement in development, as also reflected in users finding the overall program unhelpful [40]. However, another study reported that users found microinterventions helpful when experiencing an effect [54], and that reminders could act as behavioral nudges [54,70], but noted negativity toward repetition [54].

User preferences for engagement, timing, and context are reported as versatile and wide-ranging between people and even within the same individual over time, with the need for multiple levels of user control [56]. Contextual tailoring of content has also been noted to promote engagement and increased effectiveness compared to other conditions [15], highlighting a need for systems delivering contextually relevant and user-preferred content [54]. When given a choice, users seem to prefer certain interventions even if these are not the most effective [48,56]. Paradoxically, one study found that users perceive low-effort interventions as more helpful despite results indicating higher effectiveness from high-effort ones [56]. Perceived usefulness likewise seems to play an important role in attrition, as those who initially experienced larger immediate improvements were less likely to drop out [14]. Other aspects of personalization include individual versus social microinterventions to better suit user preference [15] and the need for novelty itself [56]. One study further reported that some users prefer a higher or lower number of sessions, longer or shorter training, indicating these as parameters for personalization that may increase acceptance [48].

Generally, the levels of personalization available to users across studies vary greatly; there is no one-size-fits-all intervention [15,64,89] as different users may have different preferences or needs for the level of personalization available and the extent to which they can control the system. Based on these insights and the D-MIST framework, we propose four levels for personalization that can be found in Multimedia Appendix 4 [14,15,19,48,49,56,58,60]. These may occur through the user’s control or automated system adaptation at each level: event, microintervention, narrative, or system adaptation.

#### Efficacy

In this section, we will briefly cover the effects reported by the included literature.

Interventions addressing body dissatisfaction have found both event and microintervention effects, although these report mixed results as to whether effects persist past the intervention or not [14,65,67]. Self-compassion tasks and meditation interventions, for example, both elicited positive results in body dissatisfaction [68,69]. However, no poststudy retention of these effects was found after 7 days despite consistent immediate effects [65]. Conversely, Fardouly et al [67] reported that viewing a small number of positive and neutral body social media posts not only had immediate effects but also that these were retained after 4 weeks.

In stress interventions, there are indications of positive effects after 4 weeks, for example, higher stress self-awareness and a reduction of depressive symptoms, with interventions chosen by machine learning showing better coping behaviors [15]. Momentary stress reduction effects seem persistent between delivery methods, such as prescheduled versus JIT. However, prescheduling was associated with greater advancement through behavior change [56]. Additionally, a gamified approach may be advantageous compared to other conditions in terms of effects and enjoyment [58]. Tong et al [54] found system-chosen and user-chosen microinterventions to have similar effectiveness, but note that the system-chosen provides greater variety in used intervention content. Johnson et al [55] reported a higher effect from a single 20-minute video-based microintervention compared to 16 2-minute microinterventions, although 2-minute microinterventions may be more applicable in certain contexts. Johnson et al [55] further speculate that multiple microinterventions tailored to the individual and their specific context may have additive effects related to both proximal and distal outcomes.

Momentary effects are likewise reported for mood [16,17,48,62,63], with indications that there are declines in depressive and anxiety symptoms over time, with one study noting no added benefits from JIT [62]. Momentary effects also include the microintervention’s ability to reduce the frequency of unpleasant dreams [74] and reduce social anxiety [76]. Other distal effects reported include the ability to increase physical activity [18], improve self-efficacy of parents of children with severe disabilities [38], improve meditation adherence [49], and improve systolic blood pressure [72].

## Discussion

### Principal Results

The D-MIST framework provides a useful tool for designers and researchers to design and study narratives and their effects, including opportunities for personalization of the constituent components. It can be particularly helpful when comparing different systems, when considering reuse of components from other systems, when detailing and linking components to their outcomes, and as a way to structure and describe new designs.

Compared to prior works defining microinterventions [12,14], D-MIST is the first to look at microinterventions from a system perspective and the first to define and detail how these systems operate through various interactions. D-MIST’s interaction model is additionally the first to explain how different modes of interaction with the system and the inclusion of other actors form a consolidated narrative over time. The D-MIST framework is a domain-situated framework that leaves the choice of which BCT to deploy for momentary change as a design choice, as opposed to more general DBCIs. Unlike previous intervention patterns [23,46], microinterventions operate exclusively with short events, aiming to have an immediate impact [12,14]. While these typically operate as EMI [15,18,38,48,54,56,70], there are some exceptions where these do not occur naturally in users’ daily lives [57,71]. We have also seen that microinterventions are not necessarily JIT or JITAI. The D-MIST framework, therefore, covers both microinterventions that use these patterns, for example, JIT [56,62], and those that do not, for example, on-demand [14,48,56].

The D-MIST framework allows for creating and systematically describing an overall integrative and coherent experience at all levels encountered by the user, including the narrative, microinterventions, and their connected events. The D-MIST framework collects scattered insights highlighting the role of the narrative in addressing diverse needs and preferences over time. This led to insights into actors’ roles in the creation and maintenance of narratives over time. One advantage of the clear narrative, microintervention, and event separation is that it allows for independent testing and evaluation, for example, of microinterventions [14,38,67,90] or events [49,55,58]. Individual narrative components can thus be evaluated ahead of time, providing options for improvement or selection. Tests may help explore the relevance of the microintervention to designated users and circumstances, informing conceptual models and decision rules.

### Overall Design Guidelines

#### Design Approaches

Two ways to design microintervention systems are top-down and bottom-up. In a top-down approach, designers may start by (1) considering the facilitating systems’ role, including its intended interaction model, followed by (2) considering the narrative aims and intended user experience over time, and (3) including which microinterventions and events are relevant to the desired narrative or designing new microinterventions based on the narrative needs.

We may, for example, aim to create a system that helps users sleep better (narrative goal) with an on-demand user-controlled interaction model. Initially, this could be supported by educational microinterventions and later, based on user self-identified needs, for example, reducing unpleasant dreams or reducing nightly screen time.

In a bottom-up approach, designers may start by (1) selecting, gathering, or designing a catalog of microinterventions and their events, then (2) exploring and designing various narrative experiences based on the included catalog, and finally, (3) designing how these narrative experiences may be facilitated by the system and interaction models.

When designing a sleep-improving system, designers may have a good grasp on factors affecting sleep and microinterventions that help mitigate these. Designers may desire to create a narrative experience where users are served these microinterventions based on their needs, so as not to overwhelm users with less relevant microinterventions. This could result in an interaction model where the system collects data, analyzed by clinicians who then recommend microinterventions based on identified needs.

These approaches and examples are, however, rather simplified and do not consider more iterative or user-centered approaches to design, which we highly advocate for. Given that microinterventions aim to be flexible, match user preferences, capacity for engagement, and need, there seems to be significant advantages in using more user-centered approaches to design, for example, co-design or cocreation [44], allowing cooperative design and exploration of systems and microinterventions when useful. Multimedia Appendix 5 highlights a more detailed example of a top-down approach.

In a recent co-design project, end users designed a web-based microintervention system focused on short videos providing education and presenting different approaches to self-management behaviors. Users themselves specified (in a top-down fashion) the overall system requirements, interaction model, overall narrative expectations, and even designed groups of content (events) bound together by need or topic (microintervention). Groups of educational videos were imagined providing various means of increasing physical activity, followed by groupings of the videos presenting more details on these approaches, including implementation and tips, and a discussion on their relevance (ie, the conceptual models and the narrative over time). Interestingly, neither D-MIST nor microintervention terminology was brought up by facilitating authors practicing proactive neutrality [91], nor was it explicitly used by participants in the process; however, it resulted in a system that fits the D-MIST framework.

#### System Design

The type of microintervention system heavily influences the design and implementation. JIT systems may be more challenging to develop due to the need for more explicit [73,92-94] operational conceptual models and decision rules for when to trigger specific microinterventions and events. On-demand and prescheduled systems, on the other hand, may not need to deal explicitly with conceptual models or decision rules as users themselves choose when and which microinterventions and events to engage with. Such designs, however, may consequently push larger responsibilities to the user [40,54,56], even if increasing the user’s agency. Alternatively, combining different actors in the interaction model may be considered [19,20]. Future work should explore the advantages and disadvantages of existing [56] and new interaction models.

#### Narratives

The D-MIST framework highlights the importance of the narrative structure not only in telling a meaningful story but also in ensuring that the order in which interventions are presented is meaningful to the goal, the user’s situation, and needs over time. Multimedia Appendix 3 [18,40,56,70] presents an example of a microintervention narrative aiming to improve physical activity through various microintervention goals, with Multimedia Appendix 6 presenting an example of how a monitoring-analysis-intervention approach may be used in the context of microinterventions. Designers need to consider how the narrative is presented, even if users themselves are ultimately responsible for consuming the presented events, and thus become the owners of the narrative. However, the ownership in, for example, a clinical context, could also be shared between the actors (patient and provider) [95,96]. Interactions could also be more complex, for example, targeting both couples [97,98] or adults and their dependents [99-101], where one actor may act as a medium for another. Further, it has previously been argued that users are often unable to recognize the need to engage and what support is needed [23]. This suggests that there may be advantages of increasing the systems’ ownership of the narrative [56], although recent studies contest this notion [56,62].

When building on-demand and prescheduling [56] features, designers should pay close attention to how users are expected to create the narrative, for example, by presenting microinterventions and events in the order they are to be consumed [22]. We suggest providing users with transparent information on the relevance of interventions in a given context. This may, however, pose challenges for systems with large catalogs of interventions that may need to be prioritized, grouped [22], or have the system recommend a subset of relevant interventions [15] for the user to choose between.

#### Building Microinterventions and Events

When building microinterventions, it is important for designers to consider the scope of the intervention, including the number, types, and context of events. A rule of thumb is that microinterventions and their events should be designed with the user segment in mind and aim to match their capacity for engagement [12]. Moreover, events could contain variations of exercises to engage users for longer periods [14]. Designers might also consider whether events should have a logical order to them [38,39] and whether these should have adaptive elements, for example, adjusting exercise length or resource format to match context [39,55]. Contemporary studies suggest that a need for novelty in interventions occurs rapidly [15,56], implying that most microinterventions should not be used indefinitely.

When creating events, designers should first consider the aim of the microintervention, the number of connected events, and their type. This informs the initial considerations on decision rules, including their contextual appropriateness and internal dependencies [39,56]. Designers should also carefully choose which resources best engage users’ expected contexts; for example, certain exercises may be best facilitated using audio [62], others, imagery and text [39].

Events may be designed in a number of ways; for example, Paredes et al [15] demonstrate how events may be developed from “Pop-Culture” [54,60,67]. Others adapt existing interventions like behavioral activation [4,17] and episodic future thinking [39,81,102], or are inspired by related studies [60,67]. The significant literature on BCTs and established interventions may also be of particular interest in developing new microinterventions and events.

Designers and researchers may find small trials [68,69,74] useful in determining effects and user perceptions of events, guiding the design of their decision rules. Prior works have used the Amazon Mechanical Turk for this purpose [16,17,55].

### Users’ Role and Narrative Scopes

While the currently presented systems operate primarily in the range between a day and a month, we see significant potential in systems that can deploy microinterventions for prolonged periods of time. Behavior change is well known for being challenging, often taking considerable time. Microintervention systems, by their nature, have the potential to support users with microinterventions, engaging them only when necessary and potentially reducing user fatigue and attrition, short-term, as well as long-term. For instance, a system aiming to support stress reduction through narratives may operate at a short or intermediate timeframe, while support for chronic illness may need to consider longer-term, distal perspectives. Such perspectives could, for example, take the form of a system acting as a companion, digital nurse, or facilitate microintervention delivery based on a nurse or clinician’s assessment [20]. Overall, how long a microintervention system should aim to operate is tied to the use case and overall design, including the system’s interaction model.

As we have highlighted through this paper, the interaction model can be used to describe or explain the relationship between the user, the system, and other actors. This model is particularly important as it explains a crucial part of the system, namely, how interventions are delivered to the user. We have previously noted how, for example, a narrative may be facilitated between user and clinician through the system or may be user or system-controlled. Hence, the interaction model can make such relationships more explicit. Of course, as denoted by our definition of a microintervention system, the narrative should be viewed in light of both the system design and interaction model. While the interaction model formalizes how the aims of the system are met, the design also plays a key role. The system design may, for instance, be an ordered list of microinterventions, unlocked by order of consumption, or may have the users periodically choose which microinterventions they engage with, in which case users directly engage with the narrative. Alternatively, in a JIT type system, the narrative can be hidden behind the scenes or shown to users through a history of interventions. In this way, the narrative can be seen both as a tool for design and, in other cases, as something users interact with directly.

While one of the inherent aims of microinterventions is to reduce fatigue by lowering barriers to engagement by their short nature [12], there are indications that a need for novelty can appear relatively quickly and that repetition can be seen negatively [56,70]. This may indicate that the nature of a single microintervention by itself is not enough to fully mitigate fatigue. D-MIST aims to address this by emphasizing the expectation that microinterventions are deployed with a finite lifespan and can be switched or interchanged in accordance with users’ evolving circumstances, that is, here, the need for novelty. However, user needs may also change more fundamentally over time, for instance, due to the progressive nature of type 2 diabetes, which may necessitate the deployment of more microinterventions and events over time, responding to emerging needs such as diabetes distress [78]. D-MIST’s emphasis on deploying microinterventions based on users’ circumstances and best-fitting conceptual models (ie, the available microinterventions) aims to keep the narrative coherent and thus prompt designers to more explicitly consider evolving needs over time. In the case of more user-controlled narratives, it may include features for supporting users to self-identify needs, while in other settings, it may be done through interactions with clinicians or even automatically by the system.

### Narratives for Personalization and Improved Distal Outcomes

While we have seen a few considerations for narratives leveraging the synergy between microinterventions, early results are promising. Various authors [14,17,49,55,57] note that there seem to be advantages to combining different types of interventions. Howe et al’s [56] finding that users are inclined to see low-effort interventions as more useful and Fuller-Tyszkiewicz et al’s [14] finding that perceived usefulness has an impact on dropout rates indicate that there are advantages in using low-effort interventions initially compared to more clinically relevant but intensive interventions. This further supports the notion that users may not wish to invest much effort in interventions [12]. Similarly, laboratory results suggest that a priming or educational microintervention can increase subsequent engagement [49]. Some authors likewise suggest targeting barriers stemming from stress or depression to increase subsequent engagement [57,78]. Leveraging such effects seems key to achieving distal effects while increasing engagement, reducing attrition, and ultimately improving long-term health outcomes.

However, the idea of creating personalized narratives is contingent on our understanding of the users and the building blocks we use. Most studies report positive effects [55,69,74], although varying between in-moment and more persistent effects [14,56,67]. Multiple different microinterventions can also affect the same targets, but insights into the user experience suggest users may have different personal preferences for these [40,48,56], affecting the perceived usefulness and uptake.

Digital solutions also offer benefits beyond clinician-provided care, such as constant availability [103] and discrete daily use. The availability of mobile health has caused a shift away from strictly clinical settings toward self-exploration using technology [13]. Traditionally, interventions and health care have been provided by professionals based on an understanding of the patient’s health needs, suggesting relevant interventions at their discretion. Nevertheless, research suggests patients’ experiences are not always in line with clinical judgment [104] and that limiting patients’ autonomy may hinder the exploration of self-management, impacting long-term engagement [13,78].

However, high attrition is common in digital health [6,7], with some noting a lack of rigorous study practice, clear evidence-based therapeutics, and clinical evidence [56,105,106]. Moreover, as shown by Howe et al [56], users may not always be aware of which microinterventions are the most effective in reducing symptoms. This is also supported by Van Cappellen et al [49], speculating that users’ increased engagement was caused by a psychoeducational microintervention, allowing participants to engage with meditation more effectively. More broadly, the relationship between health knowledge, preferences, and effectiveness in microinterventions has yet to be explored, presenting opportunities for future research.

Despite D-MIST being built on a review of more than 30 microintervention systems covering more than 500 unique events, we know little about each microintervention’s best use, its conceptual models, and narrative implications, with Hojjatinia et al [30] noting the need for person-specific rules. The insights we uncovered on narrative tailoring suggest it may be key to designing systems that enhance activity enjoyment, motivation, retention, and ultimately improve distal outcomes [30,49,55]. This highlights the need for more research on narratives and conceptual models, including user-centered factors.

### Limitations and Future Works

As a consequence of the early stage of research in microinterventions and our desire to create a framework rather than systematically describe the entire body of literature, we have chosen a CIS approach. The CIS method [24] allowed us to use the available evidence effectively, even if the field of research is not fully formed. While we developed and validated the framework through the CIS methodology, through the experience of the research team, and through retroactively applying D-MIST back onto the literature, more work is needed to confirm the generalizability of the framework. A draft version of the framework has been tested with engineering students and has received early feedback from some fellow researchers, but a more formal assessment of the framework has yet to be carried out. Future work, therefore, includes conducting a formal expert review of D-MIST and assessing how D-MIST is practically leveraged in the design process of new microintervention systems. The empirical work of developing D-MIST has thus far been limited to universities in Denmark and the United Kingdom; future work should therefore also aim to include experts from other contexts.

Further opportunities to extend and validate the framework include applying it to a case study aiming to look at real data, to support physical microinterventions that have a more direct impact on users, such as the deployment of medications, or to include nonintrusive short measurements of the different levels of the framework.

The D-MIST framework might additionally be useful for structuring the analysis and design related to privacy, security, and ethics, for instance, by going through components step by step and considering issues related to these topics. Considerations for events might, for example, include assessing their suitability for all targeted users (ie, universal design, inclusivity, and accessibility), and for triggers, assessing the impact of collecting past historical data and being able to track and assess the user’s current state. However, future works are needed to further explore these topics in relation to microinterventions and D-MIST.

The number of interventions, study periods, and the number of participants in many of these studies is limited. Adding additional literature not focused on microinterventions would, however, have made our work less focused. Consequently, papers describing similar systems [3,22,29,107] without using the microintervention terminology and their insights are outside the scope of this work. We also did not exclude literature based on quality, which may have resulted in the inclusion of some “lower quality works.” However, we note in line with the CIS methodology that exclusion of literature should not be based on quality grounds alone, as such literature can inform, contribute to, and add to the richness of findings [24,50,108], and hence, we did not use a structured tool for assessment.

While we have proposed an evidence-based framework (D-MIST) for microintervention systems, reasoning about scalable and engaging digital health interventions, several questions remain: first, given the relatively short duration of most microintervention studies, we know little about long-term use, distal effects, and the perspective of maintaining varied narratives in both short- and long-term. Most “long-term” studies on microinterventions are indeed relatively short compared to more traditional long-lasting behavior change studies where behaviors are to be maintained over years [109]. The broader microintervention care life cycle and user experience over time may be key to understanding the wider requirements for microintervention systems. Second, we have uncovered relatively few microinterventions with clearly scoped decision rules and use contexts, making it difficult for designers of microintervention systems to leverage existing research, especially outside the reported uses. Even though more research is needed on both individual microinterventions, on the larger narrative applications, and in the exploration of the user experience, the D-MIST framework can be a helpful tool when structuring and describing microintervention systems.

### Conclusions

Microinterventions hold the potential to engage users who would otherwise not commit to traditional interventions due to the effort required. To help the design, assessment, and scientific reasoning about microinterventions and their constituents, this paper has presented the D-MIST framework. D-MIST allows for classifying, designing, describing, and studying microinterventions, the systems providing them, and their embodied narratives. The framework is based on a review of state-of-the-art research on microinterventions and has consolidated, discussed, and specified definitions across a large body of evidence.

The framework describes microinterventions, their components, assessments, and implications for the user experience of engaging with microintervention systems. This paper also documents the need for personalization and adaptability of events and their resources and delivery method, and discusses automation versus ownership and user agency. Explicit narratives have the potential to create more meaningful, effective systems with personalized and contextually relevant microinterventions. The studies included in this review support the notion that tailoring narratives to the individual can lead to increased effectiveness, exercise enjoyment, barrier reduction, and reduced attrition. Leveraging synergy between different microintervention and their effects for use in narratives seems key to supporting better long-term health outcomes.

The D-MIST framework addresses the challenge of understanding how we can design technologies and apps that productively leverage multiple microinterventions in personalized narratives supporting not only short-term goals but also long-term health outcomes. Furthermore, the framework promotes an understanding of how different actors, collaboratively through their interactions, achieve the aims of microintervention systems.

## Appendix

Multimedia Appendix 1 Full search strategy, and database specific query, no filters or limits were applied to any database.

Multimedia Appendix 2 Examples of micro-intervention systems and their components according to the D-MIST framework.

Multimedia Appendix 3 A proposed micro-intervention narrative with a narrative goal of increasing physical activity through various micro-intervention goals, based on a combination of micro-interventions and events found in existing literature. A detailed description of the narrative, micro-intervention goals and micro-interventions can be found below. A similar timeline could be used to highlight an individual narrative thread experienced by a user by including specific events.

Multimedia Appendix 4 Opportunities for Personalization.

Multimedia Appendix 5 Step by step example of a top-down design process for a hypothetical micro-intervention system aiming to increase physical activity.

Multimedia Appendix 6 A monitoring-analysis-intervention (JIT) approach to creating personalized micro-intervention narratives.

## Abbreviations

BCT: behavior change technique
CIS: critical interpretive synthesis
DBCI: digital behavior change intervention
D-MIST: Design Framework for Microintervention Software Technology
EMI: ecological momentary intervention
JIT: just-in-time
JITAI: Just-In-Time Adaptive Intervention
PRISMA: Preferred Reporting Items for Systematic Reviews and Meta-Analyses
