# Supplementary material for: A Design Framework for Microintervention Software Technology in Digital Health: Critical Interpretive Synthesis
Source: J Med Internet Res. 2025 Sep 12;27:e72658. doi: 10.2196/72658 (PMC12475881; doi:10.2196/72658)
Supplement: Multimedia Appendix 1 [file jmir_v27i1e72658_app1.pdf]

Database	Search strategy (database query)
ACM	AllField:("micro-intervention" OR "micro intervention" OR "micro-interventions" OR "micro interventions")
Pubmed	"micro-intervention"[All Fields] OR "micro-intervention"[All Fields] OR "micro-interventions"[All Fields] OR "micro-interventions"[All Fields]
SCOPUS	TITLE-ABS-KEY ("micro-intervention" OR "micro intervention" OR "micro-interventions" OR "micro interventions")
WOS	ALL=(“micro-intervention" OR "micro intervention" OR "micro-interventions" OR "micro interventions”)
