# Supplementary figures and images for: A Design Framework for Microintervention Software Technology in Digital Health: Critical Interpretive Synthesis
Source: J Med Internet Res. 2025 Sep 12;27:e72658. doi: 10.2196/72658 (PMC12475881; doi:10.2196/72658)

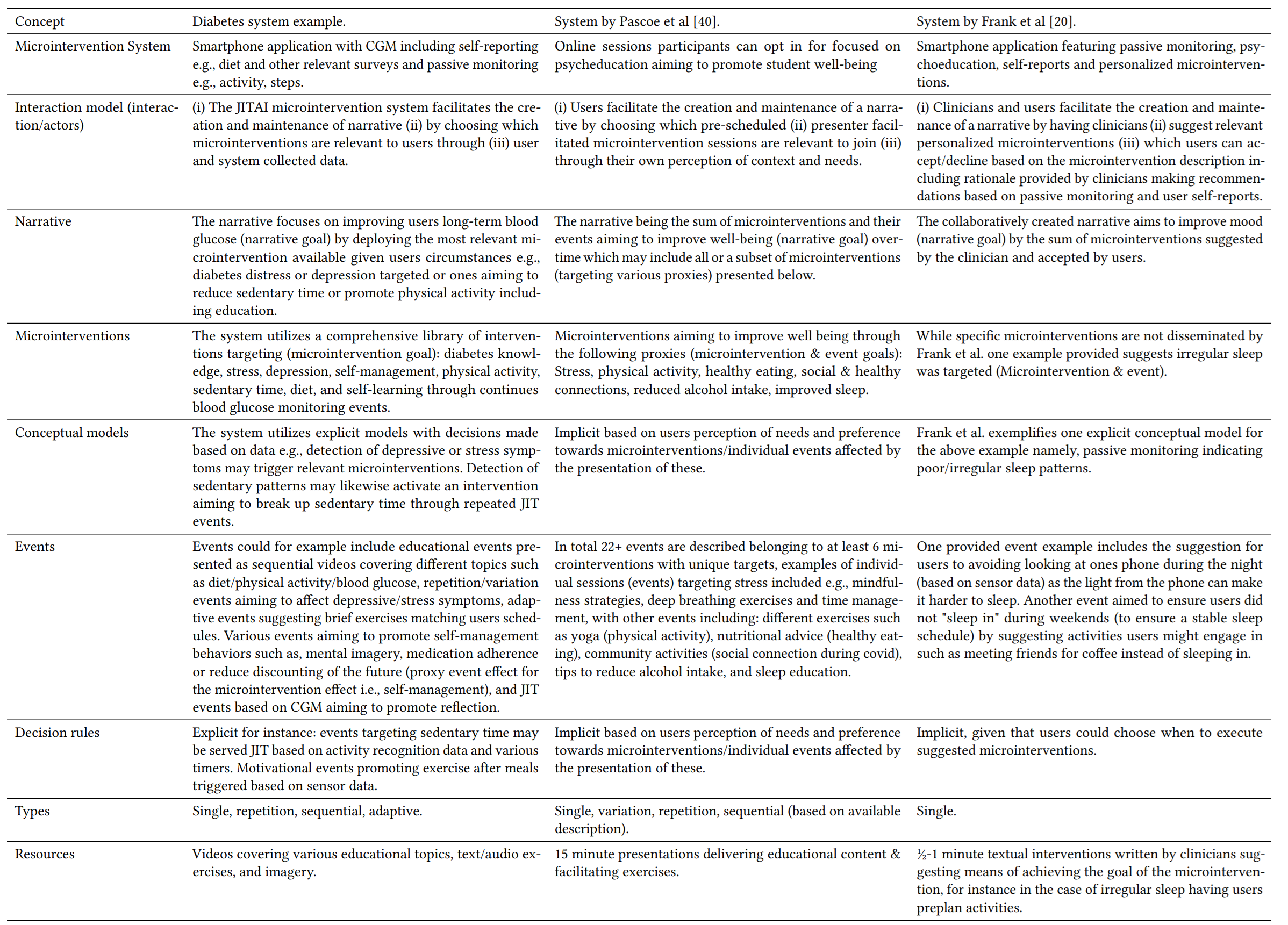

Supplement: Multimedia Appendix 2 [file jmir_v27i1e72658_app2.png]

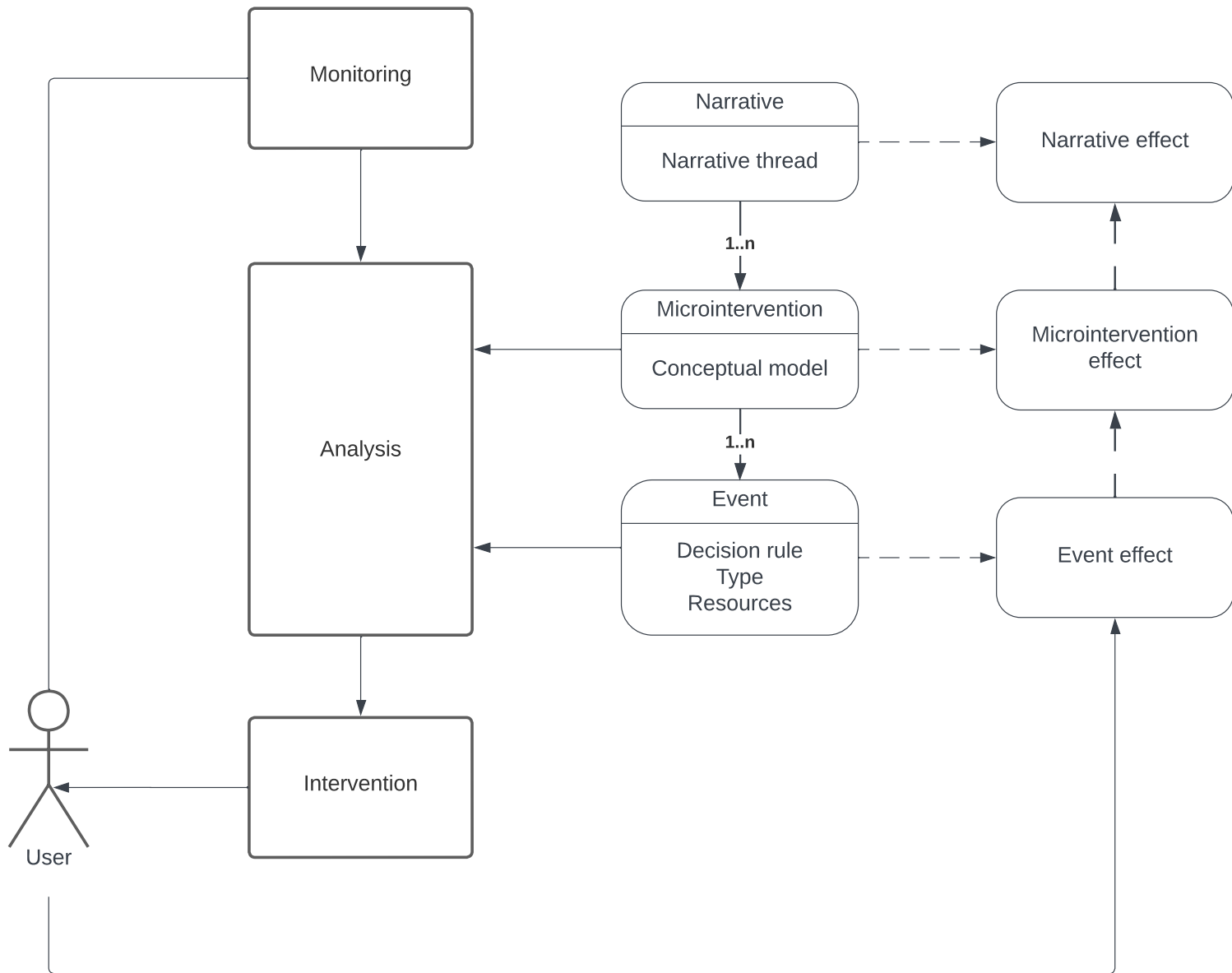

Supplement: Multimedia Appendix 6 [file jmir_v27i1e72658_app6.pdf]
