# Supplementary material for: A Design Framework for Microintervention Software Technology in Digital Health: Critical Interpretive Synthesis
Source: J Med Internet Res. 2025 Sep 12;27:e72658. doi: 10.2196/72658 (PMC12475881; doi:10.2196/72658)
Supplement: Multimedia Appendix 3 [file jmir_v27i1e72658_app3.pdf]

Microintervention Narrative (Physical Activity)

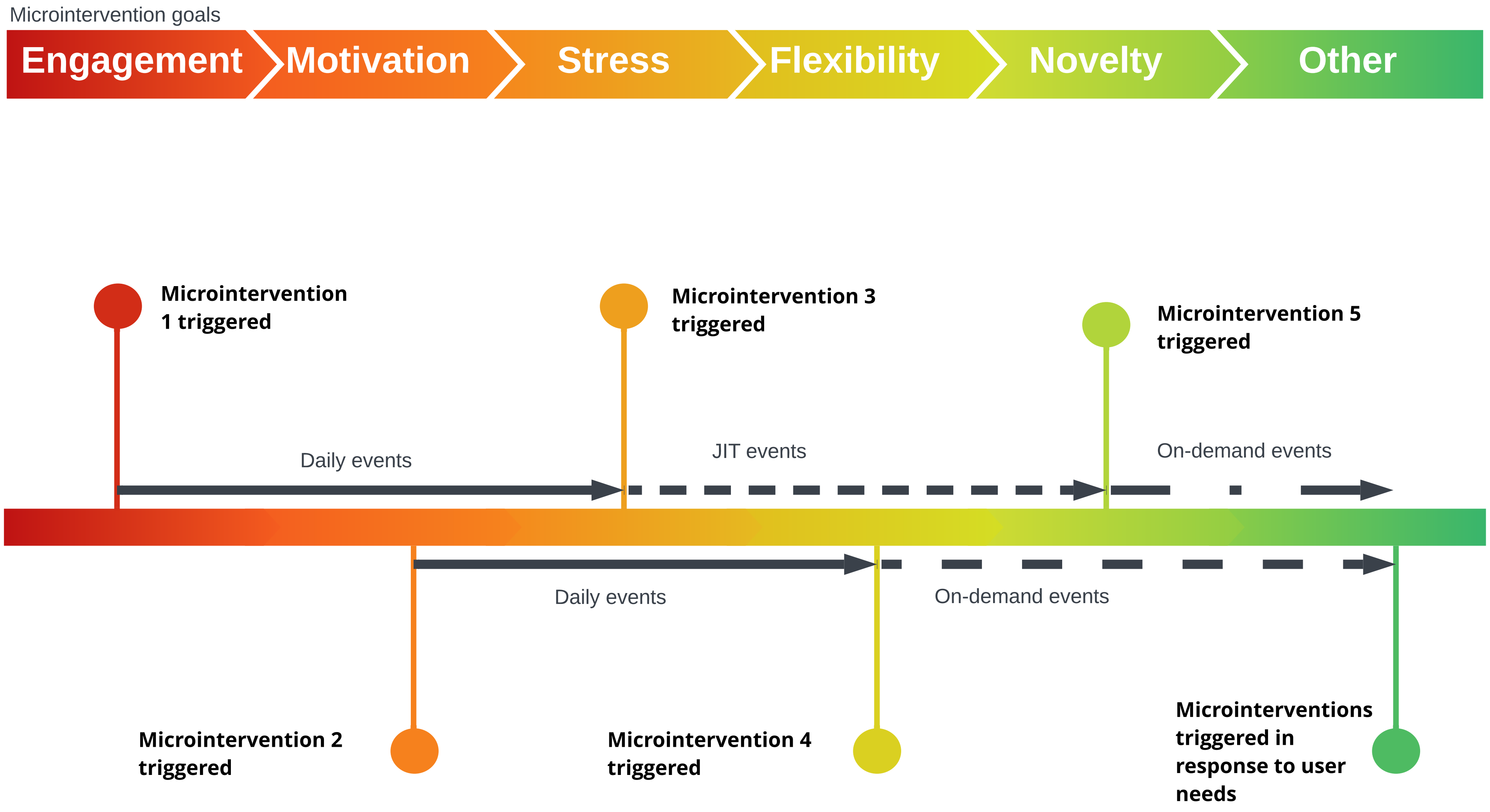

Narrative goal: Improve physical activity.

Interaction model: Users can choose between different modes of interaction with the system, in this example: JIT, pre-scheduling and on-demand.

Microintervention goal: Improve engagement
Description: Users may initially have low willingness to engage in healthy behaviors/interventions e.g., measured through high delay discounting (Persson et al.). Detection of high discounting could therefore trigger appropriate micro-interventions such as micro-intervention 1.

Microintervention goal: Motivation
Description: Users may lack knowledge or motivation to engage in small activities, that could improve physical activity. Smartphone detection of low physical activity or extended sedentary time could trigger a micro-intervention such as micro-intervention 2. However, this micro-intervention could also be triggered initially to impart knowledge and awareness.

Microintervention goal: Stress
Description: System detection of high stress could trigger micro-intervention 3 to reduce stress related barriers.

Microintervention goal: Flexibility
Description: Users may have a need for novelty or improved flexibility in micro-interventions/event timing. The system could detect and suggest prescheduling upon detecting low engagement with recommended/deployed JIT interventions or users themselves could initiate this change and then choose micro-intervention 4.

Microintervention goal: Novelty
Description: A need for novelty could similarly have users transition from other micro-interventions to a more on-demand approach where they choose when to engage with different micro-interventions such as micro-intervention 5. Users could e.g., find the messages in 2 repetitive over time or find the need for small physical activity reminders from 4 unnecessary as behaviors become more habitual.

Microintervention 1: Episodic Future Thinking Persson et al [70].

Conceptual model: 1) The user shows signs of high delay discounting lowering engagement with other interventions (e.g., measured through a delay discounting adjusting amounts taks)

Event: Self guided generation task
Event Type: Repetition
Resource: Exercise aiming to create text representing a future event as well as an audio recording of said event in the users own voice
Decision rules: 1) EFT triggered

Event: Audio Reflection task
Event Type: Repetition
Resource: Reflecting upon previously generated future events.
Decision rules: 1) One self-guided generation task completed. 2) time is before first or last meal of the day. 3) no reflection task has been undertaken in the last 6 hours. 4) User has no apprehension towards own voice.

Event: Text Reflection task
Event Type: Repetition
Resource: Reflecting upon previously generated future events.
Decision rules: 1) One self-guided generation task completed. 2) Time is before first or last meal of the day. 3) No reflection task has been undertaken in the last 6 hours. 4) User has strong apprehension towards own voice.

Event: Mental imagery reflection task
Event Type: Repetition
Resource: Image (notification) representing one of the generated future events.
Decision rules: 1) One self-guided generation task completed. 2) Time is before first or last meal of the day. 3) No imagery reflection task sent recently. 4) User is in a context where delay discounting modification is needed but other tasks are contextually inappropriate.

Microintervention 3: JIT Stress reduction Howe et al [56].

Conceptual model: 1) High stress detected. Upon decision rules being fulfilled users choose one of the following event "catagories" and is presented a variation of that event.

Event: Get my mind off work
Event Type: Repetition/variation
Resource: Chatbot suggests engaging in an activity
Decision rules: 1) a computed stress score based on five components (email volume, calendar saturation, time of day, facial expression and heart rate, or ecological momentary assessment stress score if other data is missing), 2) within weekday and working hours, 3) no other interventions scheduled later, 4) the user has not completed an intervention in the past hour, 5) no nudge has been sent in two hours, 6) and the total nudges today has not exceeded four

Event: Feel calm and present
Event Type: Repetition/variation
Resource: Chatbot facilitated mindfulness exercise
Decision rules: 1) a computed stress score based on five components (email volume, calendar saturation, time of day, facial expression and heart rate, or ecological momentary assessment stress score if other data is missing), 2) within weekday and working hours, 3) no other interventions scheduled later, 4) the user has not completed an intervention in the past hour, 5) no nudge has been sent in two hours, 6) and the total nudges today has not exceeded four

Event: Think through my stress
Event Type: Repetition/variation
Resource: Chatbot facilitated cognitive reframing exercise
Decision rules: 1) a computed stress score based on five components (email volume, calendar saturation, time of day, facial expression and heart rate, or ecological momentary assessment stress score if other data is missing), 2) within weekday and working hours, 3) no other interventions scheduled later, 4) the user has not completed an intervention in the past hour, 5) no nudge has been sent in two hours, 6) and the total nudges today has not exceeded four

Microintervention 2 Promote physical activity Conroy et al [18].

Conceptual model: 1) User has a need for reducing sedentary time or 2) needs to increase physical activity in general.

Event: Move more
Event Type: Variation (101 variations)
Resource: Text message
Decision rules: 1) time is between 8 am and 8pm 2) user has not received a message within the last hour and 3) less than 5 messages has been sent.

Event: General facts and trivia
Event Type: Variation (254 variations)
Resource: Text message
Decision rules: 1) time is between 8 am and 8pm 2) user has not received a message within the last hour and 3) less than 5 messages has been sent.

Event: Reduce sedentary time
Event Type: Variation (101 variations)
Resource: Text message
Decision rules: 1) time is between 8 am and 8pm 2) user has not received a message within the last hour and 3) less than 5 messages has been sent.

Microintervention 4 Prescheduled light physical activity (sedentary time)

Conceptual model: 1) Need for novelty or 2) more flexibility

Event: Micro-intervention session reminder
Event Type: Single
Resource: Exercise suggestion or self-generated
Decision rules: Implicit user pre-schedules exercises

Microintervention 5 Physical activity guidance Pasco et al [40].

Conceptual model: Implicit user may choose to engage with this micro-intervention and its events at their own leisure

Event: Physical activity guidance and sessions, delivered in the home setting e.g. aerobic exercises, yoga, high-intensity interval training
Event Type: Variation
Resource: Online video call
Decision rules: Implicit user chooses which sessions (events) are relevant to participate in
