# Supplementary material for: A Design Framework for Microintervention Software Technology in Digital Health: Critical Interpretive Synthesis
Source: J Med Internet Res. 2025 Sep 12;27:e72658. doi: 10.2196/72658 (PMC12475881; doi:10.2196/72658)
Supplement: Multimedia Appendix 4 [file jmir_v27i1e72658_app4.pdf]

At the most basic level of personalization, we see the users' ability to influence the events they engage with directly [14, 48, 56]. This could be through the devices they use as part of the system [58, 60], the meta rules of the system affecting the delivery of events [56] and more basic forms of event personalization such as the timing [15] through decision rules or the resource format. Microinterventions, their events and resources may also be adapted to better suit users based on their needs, traits or preferences [15]. Narrative adaptation meanwhile involves combining microinterventions [49] in the best possible way to support overall goals through the available conceptual models. Lastly, we see system adaptation as adapting interaction models i.e., the interactions [56] and actors [19] involved to better suit the users while adhering to the goals of the system. Thus options for adaptation may include:

- Event adaptation: Allows for basic adaptation of events, such as: 1) meta rules affecting the delivery of events such as the periods in which the system should be active or for instance which devices it should use (e.g., not all users may have smartwatches for data-collection or event delivery). 2) The timing or variation of events served through decision rules based on for instance context, needs, and personal preference. 3) The resource format served may be adapted to a given context for instance in some cases audio-based resources may not be contextually relevant or acceptable to users in general while text might be.

- Microintervention adaptation: Microintervention adaptation refers to the content served, the intensity, the timing of different events and how these are used short term. The content presented by different events may be synergized to maximize short term engagement through the number or intensity of events served aiming to match users capacity for engagement and needs. This adaption may include the use of different types of events, content, and therapy groups.

- Narrative adaptation: Narratives are adapted based on conceptual models, determining when/where to use an intervention. Each intervention choice is based on the best narrative fit. Interventions are chosen based for example on user needs, traits, and preferences. A user's personality trait or temperament may make one microintervention preferable over another. The resulting narrative thread aims to leverage the synergy between microinterventions for greater effect or by targeting various direct or indirect factors affecting outcomes or efficacy of subsequent interventions, e.g., targeting depressive symptoms and thus increasing uptake of other microinterventions.

- System adaptation: System adaptation broadly refers to users' ability to affect or personalize the interaction model. For instance users may prefer different levels of agency in the creation of narratives etc. requiring complex control over how in practice the aims of the system are achieved through the interactions and actors involved.
