# Supplementary material for: A Design Framework for Microintervention Software Technology in Digital Health: Critical Interpretive Synthesis
Source: J Med Internet Res. 2025 Sep 12;27:e72658. doi: 10.2196/72658 (PMC12475881; doi:10.2196/72658)
Supplement: Multimedia Appendix 5 [file jmir_v27i1e72658_app5.pdf]

In this example we create a simple microintervention system top-down aiming to help users reduce sedentary time and improve their physical activity, in this case focused on walking/running.

System & interaction model:
From the literature we know various microinterventions/events can be deployed aiming to educate or motivate users through microinterventions. We nevertheless also wish to create a system which directly calls users to action at opportune moments for light physical activity and supports users in improving existing physical activity. As such the **microintervention system** is a mobile application which uses builtin sensors to trigger decision rules for specific microintervention events.

With a rough outline of the system we may consider the interaction model. While it could certainly be useful to involve actors such as e.g., a personal trainer or clinician if exercise is related to lifestyle changes, their inclusion in this application is not feasible. Instead we opt to focus on an **interaction model** consisting of only the system and the user. The system will provide users with knowledge and education, and also trigger microinterventions and events based on sensor input and historical data.

Narrative aim & UX over time:
Our **narrative** aims to address the goal of increasing users' daily physical activity through a number of different microinterventions and their events.

Initially, we may assume users' need 1) knowledge and motivation to get started, 2) initial support with various types of physical activity, 3) calls to action breaking up sedentary time or suggestions for opportunistic light physical activity. As users begin carrying out more physical activity, we expect needs to switch towards: 4) carrying out physical activity at a meaningful intensity, and 5) ensuring stable physical activity.

Microinterventions & events:
Addressing these needs through **microinterventions**, we may imagine: 1) is addressed by microintervention a) consisting of **events** delivering video **resources** aiming to provide educational and motivational content. 2) is addressed by b) a single microintervention with both varied and sequential **type** events. Users can choose to watch various event videos introducing various methods of increasing physical activity, walking in nature, interval walking, running on a treadmill, urban or park running, should users be interested in one of the varied approaches they are able to watch a further sequential video on the method. 3) is addressed by c) microintervention which activates various events based on users activity as detected by mobile sensing aiming to e.g., break up sedentary time or suggest opportunistic exercises. 4) is addressed by microintervention d) consisting of sequential and adaptive events, first users choose their preferred means of exercise (e.g., running or interval walking) and a small exercise test is administered, future sequential events using this test as a basis for suggesting adjustments to users exercise intensity. 5) is addressed by e) microintervention passively

monitoring physical activity once established, triggering only occasional reminders and motivational events to promote physical activity when users are below their average.

For the sake of example, let's further break down the microintervention related to c) into a few event suggestions. Based on sensor data we might imagine an event suggesting users get up and move around after a prolonged period of sitting down (e.g., a **decision rule** of 45 min of sitting). Alternatively with more advanced feature analysis we might find users often use elevators between floors with an event suggesting users to use stairs instead of elevators for short trips or for users to get off the bus one stop earlier for a short walk.

In this example the narrative is mostly predetermined by the design of the system along with simple **conceptual models**, e.g., once users have consumed microinterventions related to needs 1), b) is unlocked, with the remaining ones subsequently being unlocked over time. The **narrative thread** in this case is thus constituted by microinterventions related to a) to e) and their events over time.

For the sake of example: If we changed the interaction model such that the narrative is user-controlled, e.g., users can freely choose between microinterventions a) to e) from the start, we might see varied narrative threads emerge between users. Some users may indeed use a) to e) in order, while others may skip a) to c) having already established preferences, and therefore exclusively use d) and e), of course some users might eventually look for alternative means of exercise leading to a thread including b).
